# Chemical Blockage of the Mitochondrial Rhomboid Protease PARL by Novel Ketoamide Inhibitors Reveals Its Role in PINK1/Parkin-Dependent Mitophagy

**DOI:** 10.1021/acs.jmedchem.2c01092

**Published:** 2022-12-21

**Authors:** Edita Poláchová, Kathrin Bach, Elena Heuten, Stancho Stanchev, Anežka Tichá, Philipp Lampe, Pavel Majer, Thomas Langer, Marius K. Lemberg, Kvido Stříšovský

**Affiliations:** † Institute of Organic Chemistry and Biochemistry of the Czech Academy of Science, Flemingovo n. 2, Prague 160 00, Czech Republic; ‡ Department of Molecular Genetics, Faculty of Science, Charles University, Viničná 5, Prague 128 44, Czech Republic; § First Faculty of Medicine, Charles University, Kateřinská 32, Prague 121 08, Czech Republic; ∥ Institute for Genetics and Cologne Excellence Cluster on Cellular Stress Responses in Aging-Associated Diseases (CECAD), Medical Faculty, University of Cologne, Joseph-Stelzmann-Strasse 52, Cologne 50931, Germany; ⊗ Center for Molecular Medicine (CMMC), Medical Faculty, University of Cologne, Joseph-Stelzmann-Strasse 52, Cologne 50931, Germany; # Max-Planck-Institute for Biology of Ageing, Joseph-Stelzmann-Str. 9b, Cologne 50931, Germany; ∇ Center for Molecular Biology of Heidelberg University (ZMBH), DKFZ-ZMBH Alliance, Im Neuenheimer Feld 282, Heidelberg 69120, Germany; ○ Center for Biochemistry and Cologne Excellence Cluster on Cellular Stress Responses in Aging-Associated Diseases (CECAD), Medical Faculty, University of Cologne, Joseph-Stelzmann-Strasse 52, Cologne 50931, Germany

## Abstract

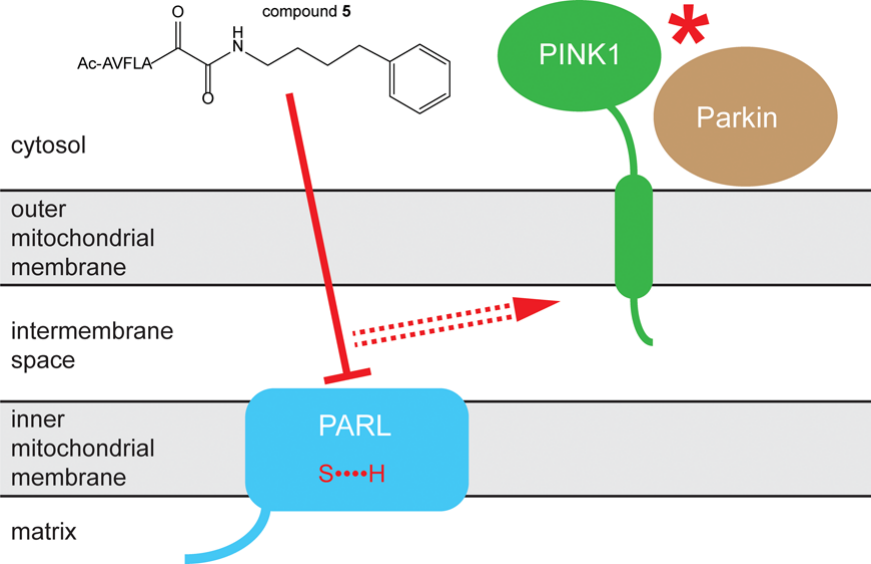

The mitochondrial rhomboid protease PARL regulates mitophagy by balancing intramembrane proteolysis of PINK1 and PGAM5. It has been implicated in the pathogenesis of Parkinson’s disease, but its investigation as a possible therapeutic target is challenging in this context because genetic deficiency of PARL may result in compensatory mechanisms. To address this problem, we undertook a hitherto unavailable chemical biology strategy. We developed potent PARL-targeting ketoamide inhibitors and investigated the effects of acute PARL suppression on the processing status of PINK1 intermediates and on Parkin activation. This approach revealed that PARL inhibition leads to a robust activation of the PINK1/Parkin pathway without major secondary effects on mitochondrial properties, which demonstrates that the pharmacological blockage of PARL to boost PINK1/Parkin-dependent mitophagy is a feasible approach to examine novel therapeutic strategies for Parkinson’s disease. More generally, this study showcases the power of ketoamide inhibitors for cell biological studies of rhomboid proteases.

## Introduction

Rhomboid intramembrane proteases play important physiological roles in eukaryotic cells ranging from signaling to protein degradation.^1−3^ They have been associated with pathological processes or pathologies including Parkinson’s disease,^4−7^ cancer,^8^ malaria,^9,10^ toxoplasmosis,^11−13^ or infection by *Aspergillus fumigatus*.^14,15^ Rhomboid proteases are thus potential novel drug targets.^16^ The most promising class of inhibitors that are currently available for rhomboid proteases are peptidyl ketoamides, which are active-site directed, covalent, and reversible,^17^ and start being used as tools in probing the cell biological mechanism of rhomboid-dependent processes.^18,19^ However, the development of specific rhomboid protease inhibitors is nontrivial without robust in vitro assays and recombinant enzymes,^19^ as is the case for a number of eukaryotic rhomboid proteases. Their development is hampered by the inherent complexities of the system such as the transmembrane nature of rhomboid proteases and their optional requirement for stabilization by their native lipid environment.^20−22^ Indeed, this is a general problem: eukaryotic membrane proteins are notoriously difficult to work with because their activity and conformation are often intimately linked to their interaction with the lipid bilayer. Their solubilization from the native membranes into surrogate environments based on detergents is empirical, and it is unpredictable whether a given protein will maintain its native properties in a given detergent. It is thus highly desirable to work with eukaryotic membrane proteins in vitro directly in lipid membranes. Although efficient fluorogenic transmembrane substrates are available for rhomboid proteases,^23^ those require solubilization of the enzyme in detergent micelles or co-reconstitution into liposomes.^23,24^ Transmembrane substrates do not spontaneously integrate into lipid vesicles and as such have limited utility for lipid-embedded rhomboid proteases, such as those produced by polymer-encased lipid nanodiscs.^25^ Here, we bridge this gap by developing small and soluble fluorescent substrates that interact only with the active site of rhomboid protease and do not require integration into the lipid bilayer. We show that these substrates are compatible with both detergent-solubilized and lipid-integrated rhomboid proteases.

The mitochondrial rhomboid protease PARL has been implicated in the maintenance of the mitochondrial respiratory chain,^26^ control of lipid transport,^27^ apoptosis,^28^ mitochondrial stress response, and mitophagy.^4−7,29^ Mutation of regulatory phosphorylation and cleavage sites has been linked to Parkinson’s disease^4,30^ although the genetic link of this mutation to disease progression has been discussed controversially.^31^ Several studies have shown that under physiological conditions, the PARL-catalyzed cleavage of a mitochondrial PINK1 import intermediate triggers its release into the cytoplasm and subsequent proteasomal degradation.^5,7,32,33^ However, other proteases have also been implicated in PINK1 processing,^32,34−36^ and PARL knockout cells have been reported to adapt by enhancing this alternative processing.^5,6^ Under mitochondrial stress such as carbonyl cyanide *m*-chlorophenyl hydrazone (CCCP) treatment, PARL cleaves the inner mitochondrial membrane (IMM) protein PGAM5 instead.^29,36^ In the absence of the PARL cleavage, full-length PINK1 then accumulates at the outer mitochondrial membrane (OMM) where it undergoes autophosphorylation and recruits the E3 ubiquitin ligase Parkin, which sets off mitophagy (for review, see ref (37)). The knockdown of PARL mimics this CCCP-induced PINK1 stabilization at the OMM.^7,33^ The exact fate of uncleaved PINK1 at the IMM and how activity of PARL can be modulated to fine-tune PINK1/Parkin-dependent mitophagy are unclear. To investigate these questions, specific acute chemical inhibition of PARL would be particularly powerful as it could side-step the adaptation that may accompany the genetic deficiency of PARL,^5,6^ but the absence of specific and potent PARL inhibitors has been a major hindrance in this direction.

Here, we use pharmacological inhibition of PARL to illuminate its role in activating PINK1/Parkin-dependent mitophagy. We apply in vitro translation in the presence of liposomes to produce recombinant PARL and use a novel in vitro assay to develop ketoamide inhibitors of PARL active in cells. Pharmacological inhibition of PARL by a ketoamide inhibitor recapitulates the major effects of PARL knockdown observed previously:^29,33^ it stabilizes PGAM5 under mitochondrial stress, it stabilizes PINK1 at the OMM, and it triggers Parkin recruitment to the mitochondria. However, pharmacological inhibition of PARL also causes alternative PINK1 cleavage and trafficking. MPP-cleaved PINK1 accumulates within the mitochondria, resembling phenotypes previously observed in PARL knockout cells.^5^ Furthermore, we observed that PINK1 accumulation in the presence of PARL inhibitors triggers its partial degradation by the IMM metalloprotease OMA1. Overall, our study reveals that chemical inhibition of PARL by ketoamides is a promising approach, yielding a novel avenue for specifically and acutely modifying the PINK1/Parkin-mediated mitophagy in health and disease.

## Results and Discussion

### Peptide Derived from the P5-P1 Region of a Transmembrane Substrate of a Rhomboid Protease Yields a Soluble Fluorescent Substrate that Does Not Require Partitioning into the Lipid/Detergent Phase

Peptidyl ketoamides interact with the active site of rhomboid proteases, covalently and reversibly binding to the catalytic serine,^17^ mimicking the substrate. They are currently the most promising class of rhomboid protease inhibitors that offer high potency and selectivity.^17,19^ However, ketoamide inhibitors of major eukaryotic rhomboid proteases of interest are scarce, particularly because of their limited availability in recombinant forms and lack of suitable in vitro assays. Recombinant rhomboid proteases are often unstable in detergent micelles and may need to be stabilized by the lipid environment.^20−22,25^ We thus sought an assay system that would be compatible with rhomboid proteases embedded both in detergent micelles and in liposomes.

**Figure 1:**
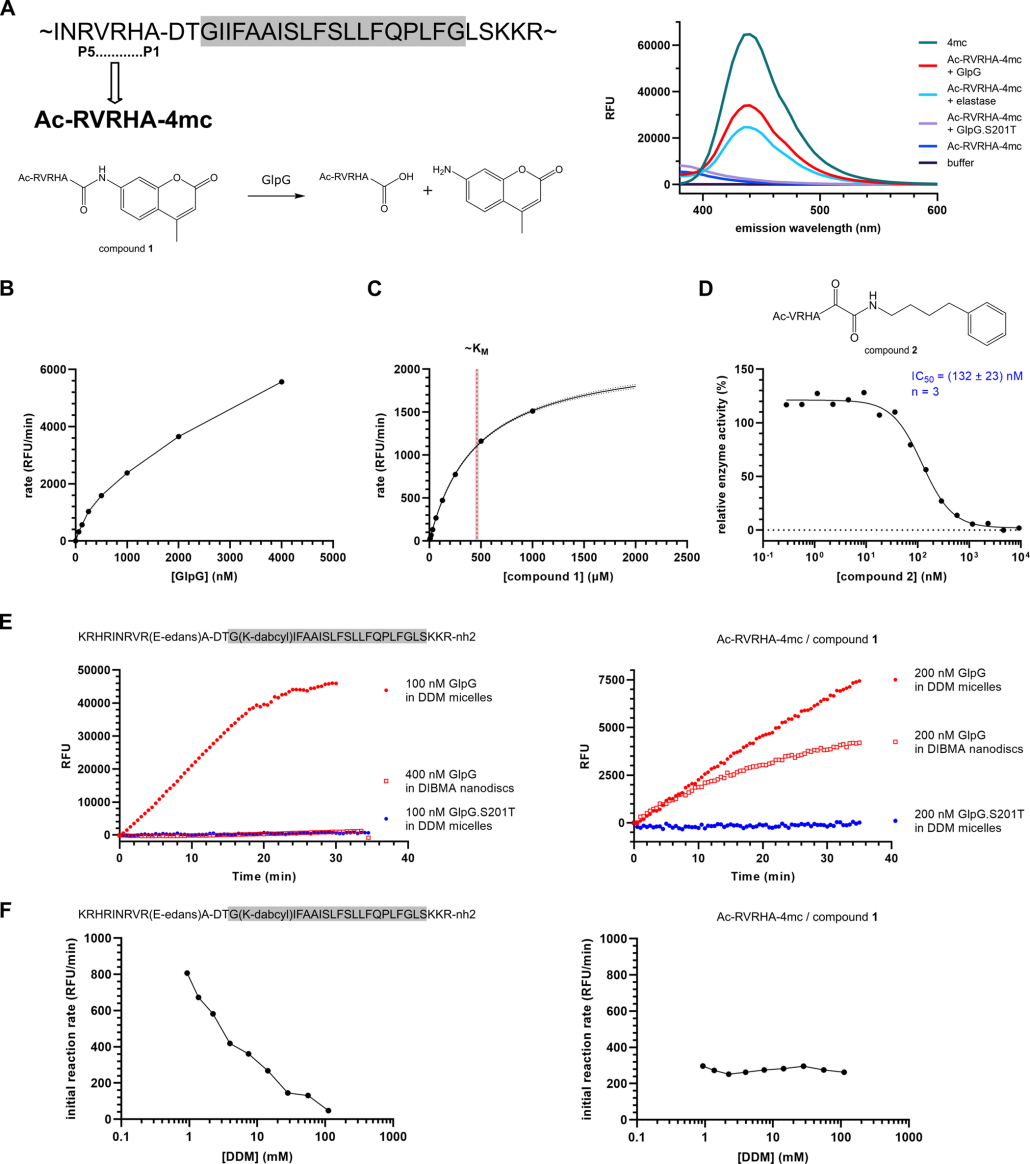
Peptides derived from the P5-P1 regions of transmembrane substrates of rhomboid proteases yield soluble fluorescent substrates that do not require partitioning into the lipid/detergent phase. Short, hydrophilic fluorescent peptide substrates can be derived from the P5-P1 region of natural rhomboid substrates. These do not require partitioning into the membrane and are thus suitable for use with lipid-integrated rhomboids. (A) Pentapeptides covering the P5-P1 region of a transmembrane substrate (whose transmembrane region is depicted by the gray background) with a preferred sequence (RVRHA) of *E. coli* GlpG^17,38^ has been modified at the C-terminus by 7-amino-4-methylcoumarin (4mc). The resulting substrate compound **1** (AcRVRHA-4mc) is cleaved by GlpG but not by its catalytic mutant to release free 4mc, leading to fluorescence increase at 450 nm. (B) Compound **1** is cleaved by GlpG in a concentration-dependent manner in the detergent micelle environment. (C) Apparent Michaelis constant for substrate compound **1** is approximately 500 μM in the DDM micelle environment. (D) Assay employing substrate compound **1** allows accurate measurements of IC_50_ of rhomboid-specific inhibitors, such as the depicted compound **2**. Enzyme concentration was 100 nM. An example inhibition curve is shown, and the IC_50_ determined from three independent measurement is shown as mean ± standard error of the mean (SEM). (E) A transmembrane substrate of GlpG (KSp96^17^) is cleaved efficiently only in detergent micelles and not in polymer-induced lipid nanodiscs formed by DIBMA,^41^ while substrate compound **1** is cleaved by GlpG with similar efficiency in lipid nanodiscs and in detergent micelles. The transmembrane region of the original substrate is indicated by a gray background. (F) Increasing concentration of DDM (micelles) induces a decrease in the initial reaction rate of cleavage of the transmembrane substrate by GlpG (transmembrane region denoted by a gray background), as described previously,^23^ while it has no influence on the initial reaction rate of cleavage of the nontransmembrane substrate compound **1**. This is consistent with compound **1** not interacting with DDM micelles appreciably.

We first focused on the model rhomboid protease GlpG from *E. coli*, whose substrate specificity has been relatively well understood.^17,38^ We designed a short hydrophilic fluorescent substrate derived from the P5-P1 region of the sequence (RVRHA) preferred by GlpG,^17,38^ modified at the C-terminus by 7-Amino-4-methylcoumarin (AMC), yielding compound **1** (AcRVRHA-4mc) (Figure 1A). Compound **1** can be cleaved by GlpG to release the aminomethylcoumarine moiety (Figure S1), leading to an increase in the fluorescence of AMC (Figure 1A) in a time and concentration-dependent manner (Figure 1B,C). The Michaelis constant *K*_M_ for substrate compound **1** is about half millimolar (Figure 1C), but the substrate is sufficiently soluble to be useable at hundreds of micromolar concentrations (data not shown). Titration of detergent-solubilized GlpG by peptidyl ketoamide inhibitor compound **2** in the presence of a constant concentration of substrate compound **1** yields the anticipated sigmoidal dose–response curve (Figure 1D), with an IC_50_ comparable to the one reported earlier considering the different enzyme concentration and different substrates used.^17^ Notably, substrate compound **1** can be cleaved by active GlpG embedded in polymer nanodiscs (Figure 1E), which is highly advantageous since their application is a convenient arising alternative to detergent systems^25,39,40^ (Figure 1E). In contrast, a transmembrane substrate^23^ cannot be cleaved in the same nanodisc system. As expected, the increasing concentration of DDM micelles leads to a decrease in the initial reaction rate of cleavage of the transmembrane substrate by GlpG, as described previously,^23^ while it has no influence on the initial reaction rate of cleavage of the nontransmembrane substrate compound **1**. This indicates that compound **1** does not interact with DDM micelles appreciably (Figure 1F) and thus represents an advantageous simplified system for the enzyme kinetics of rhomboid proteases. In conclusion, a peptide derived from the P5 to P1 region of a GlpG substrate does not require partitioning into the membrane and is thus suitable for use with lipid-integrated rhomboid proteases. We next tested this principle with recombinant PARL.

### Preparative In Vitro Translation Yields Membrane-Embedded Active PARL

**Figure 2:**
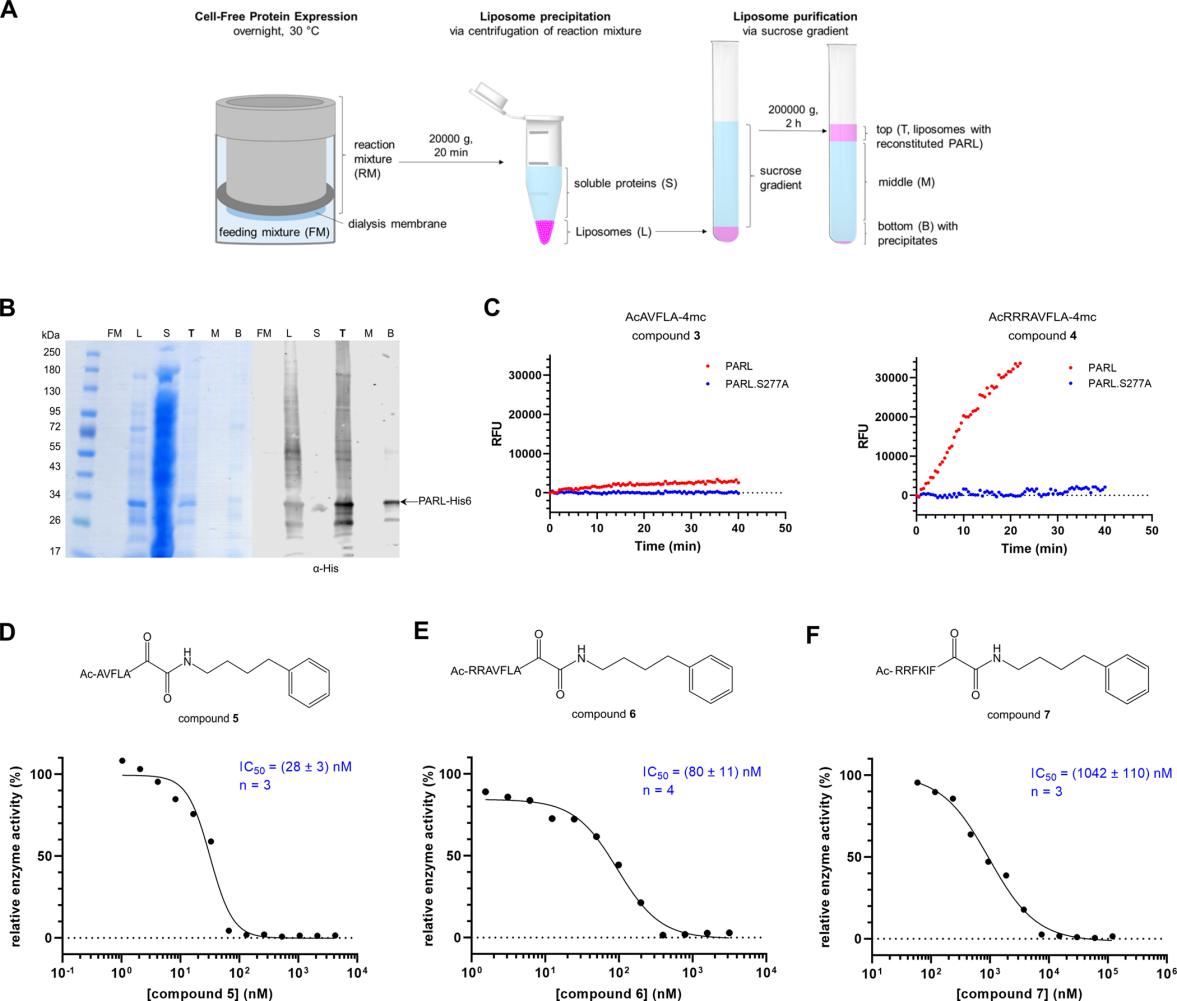
Development of in vitro assay and ketoamide inhibitors of human mitochondrial rhomboid protease PARL (A) Schematic depiction of the workflow for the production of functional PARL by in vitro translation in the presence of liposomes, PARL encoding DNA constructs are translated and spontaneously inserted into the liposomes. Resulting proteoliposomes are then isolated by density gradient centrifugation. (B) Resulting liposome-integrated PARL forms a major fraction of the isolated protein, as documented by a Coomassie-stained SDS PAGE (left panel) and anti-His immunoblot (right panel). (C) Fluorogenic substrate compounds **3** and **4** derived from the human PINK1 sequence at the PARL cleavage site^6^ are cleaved by liposome-embedded PARL but not its catalytic mutant. Compound **4**, modified for better solubility, is a more robust substrate than compound **3**. It should be noted that the ordinates in both graphs are purposefully set to an identical range so that the comparison in cleavage rates between the two substrate variants is intuitively facile. (D) Using the in vitro assay and recombinant PARL, the peptidyl ketoamide inhibitor^17^ has been derived from the human PINK1 sequence at the PARL cleavage site,^6^ yielding compound **5**. Inhibition of recombinant PARL was measured in vitro using 100 μM substrate compound **4** (Ac-RRRAVFLA-4mc). (E) To increase its solubility, compound **5** was N-terminally tagged by arginines to yield compound **6**. Inhibition of recombinant PARL was measured in vitro using 100 μM substrate compound **4**. (F) Published sequence preferences of PARL revealed by a combinatorial peptide library were incorporated into the ketoamide inhibitor (compound **7**). Inhibition of recombinant PARL was measured in vitro using 100 μM substrate compound **4**. (D–F) Mean values of IC_50_ and their SEM gained from three or four independent measurements are displayed with each IC_50_ curve.

It has been previously shown that cotranslational spontaneous folding in the presence of lipid membranes of a suitable composition can produce functional α-helical membrane proteins.^42−44^ Specifically, *E. coli* GlpG^44^ and human PARL^27^ can be produced in this way in an active form. Neither of the two rhomboid enzymes produced by cell-free translation were extensively characterized enzymatically, and we hence aimed to leverage this procedure for the development of specific PARL inhibitors. Mature human PARL (devoid of the mitochondrial targeting peptide, thus starting at amino acid 53, also known as the α-cleaved form^45^) was in vitro-translated in the presence of large unilamellar vesicles (LUVs) consisting of lipids mimicking the IMM based on the reported conditions,^27^ using a custom-made apparatus (Figure 2A). The resulting proteoliposomes were isolated by sucrose gradient centrifugation, and the presence of PARL was analyzed by SDS PAGE and immunoblotting (Figure 2B). Analogous to the approach presented in Figure 1, we generated a potential substrate for PARL by using the P5 to P1 sequence of its endogenous substrate PINK1,^6^ yielding compound **3** (AcAVFLA-4mc) and a potentially more soluble variant thereof, compound **4** (AcRRRAVFLA-4mc) (Figure 2C). Incubation of membrane-embedded PARL with substrate compounds **3** and **4** led to an increase in AMC fluorescence in a time-dependent and enzyme-concentration-dependent manner, while liposomes containing the catalytic mutant S277A of PARL maintained background fluorescence (Figure 2C). This indicated that PARL generated by the in vitro translation was catalytically active, and the assay was detecting PARL activity specifically. Substrate compound **4** showed better solubility and similar kinetics of cleavage as compound **3** and was therefore used henceforth. This result also suggests a more general way of creating in vitro assays for rhomboid proteases.

### Substrate-Derived Sequences Yield Efficient Ketoamide Inhibitors of PARL

To develop ketoamide inhibitors^17^ of PARL we followed two strategies. First, we used the P5 to P1 sequence of human PINK1, which was used for substrate compound **3**, and converted this to a ketoamide inhibitor equipped with a phenylbutyl substituent at the amidic nitrogen, based on our prior work,^17,19^ to yield compound **5** (Figure 2D). In parallel, we synthesized a variant of compound **5** (referred to as compound **6**) harboring two arginine residues at the N-terminus to potentially increase its solubility, which proved beneficial in substrate compound **4** (Figure 2E). In the second strategy, we exploited a recently reported analysis of substrate preferences of the shorter form (delta 77, i.e., β-cleaved one^46^) of recombinant human PARL purified from *Pichia pastoris*, inferred from the peptide library cleavage,^47^ which reported that this form of PARL differs from several other studied rhomboid proteases in its preference for bulky hydrophobic side chains in the P1 position, such as phenylalanine.^20^ Since it has been previously shown for bacterial rhomboids that the P1 to P5 preferences in rhomboid substrates are additive,^17,23,48^ we exploited the reported sequence preference logos of detergent-solubilized PARL reconstituted in liposomes^20^ and synthesized inhibitor compound **7** (Figure 2F), equipped with the identical warhead of compounds **5** and **6**. The in vitro assay with substrate compound **4** revealed that compounds **5** and **6** were potent inhibitors of mature PARL (IC_50_ values of 28 and 80 nM, respectively), while compound **7** was a relatively weak inhibitor (IC_50_ of 1042 nM) (Figure 2D–F). Since the AMC substrate corresponding to the sequence of compound **7** (designated substrate compound **8**) was not cleaved by mature PARL reconstituted in liposomes (data not shown), we concluded that in its combined form, the consensus sequence reported^20^ is not preferred by mature PARL.

### Peptidyl Ketoamide Inhibitor Impedes Mitochondrial Stress-Induced Cleavage of PGAM by PARL in Cells

**Figure 3:**
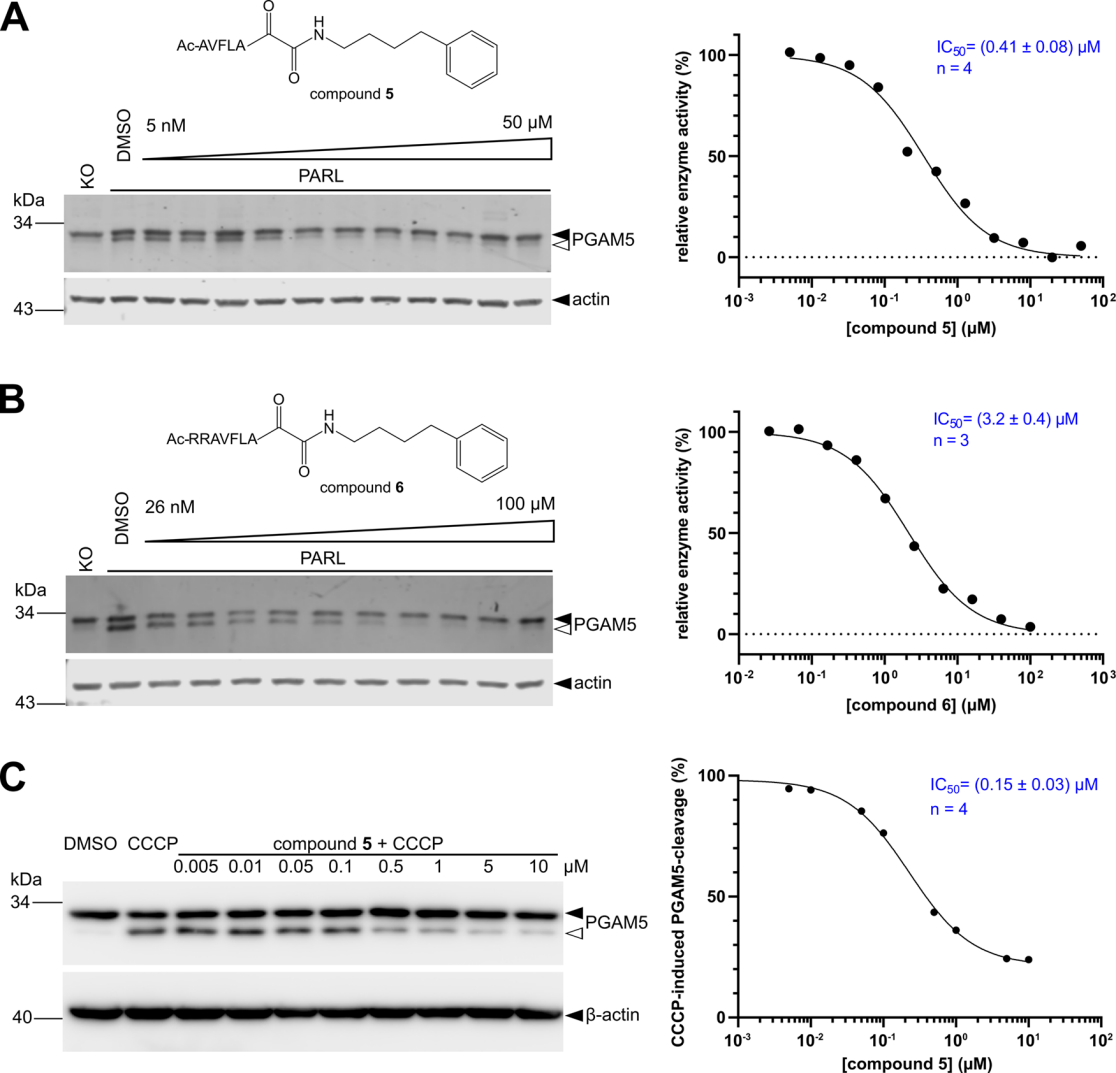
Peptidyl ketoamides inhibit PGAM5 cleavage in cells. Compound **5** (A) and **6** (B) inhibit the cleavage of overexpressed human PGAM5 in HEK293T cells in a dose-dependent manner. HEK293 T-REx PARL knockout (KO) cells stably transfected with tetracycline inducible PARL-FLAG were transiently transfected with PGAM5-Myc and analyzed as uninduced PARL KO cells, PARL-induced and DMSO-treated cells, or PARL-induced and compound-treated cells. The ratios of steady-state levels of full-length (black triangle) versus PARL-cleaved (white triangle) PGAM5 were quantified by immunoblotting. The resulting representative IC_50_ curves corresponding to the immunoblots and mean IC_50_ values ± SEM from four or three such independent experiments are displayed. Actin was used as a loading control. (C) CCCP-induced cleavage of full-length PGAM5 (black triangle) is prevented by the addition of compound **5** in a dose-dependent manner. HEK293T cells were transfected with PGAM5-FLAG, treated for 3 h, and analyzed via immunoblot. For quantification, PARL-cleaved PGAM5 (white triangle) was measured as the percentage of total PGAM5 and normalized to the DMSO condition as zero cleavage and CCCP conditions as complete cleavage. The resulting representative IC_50_ curve and mean IC_50_ value ± SEM from four independent experiments are displayed. β-actin was used as a loading control.

The nanomolar potency of the inhibitor compounds **5** and **6** in vitro prompted us to examine their effects in human tissue culture cells. Compounds **5** and **6** inhibited the cleavage of human PGAM5 by PARL overexpressed in Flp-In HEK293 T-REx cells with apparent IC_50_ values of 0.41 and 3.2 μM, respectively (Figure 3A,B). This result suggested that the N-terminal arginines, by whose presence compound **6** differed from compound **5**, may have compromised its membrane permeability, since compound **5** performed about 8-fold better than compound **6** in cells, while it was only about 3-fold better than compound **6** in vitro (Figure 2D,E). We hence focused only on the best inhibitor, compound **5**, and investigated its efficiency in HEK293T cells with endogenous PARL levels while using CCCP to induce mitochondrial stress and PARL-catalyzed PGAM5 cleavage. Satisfyingly, we found that compound **5** also potently inhibits the PGAM5 cleavage under these conditions, leading to an apparent IC_50_ value of 0.15 μM (Figure 3C). Since we observed that the inhibition of PARL by compound **5** is robust, we next used it to investigate the PINK1/Parkin pathway initiating mitophagy, in which PARL has been implicated as a key regulator.^5,7,33^

### PARL Inhibitor Stabilizes PINK1 and Reveals Alternative Cleavage Events and Submitochondrial Trafficking

**Figure 4:**
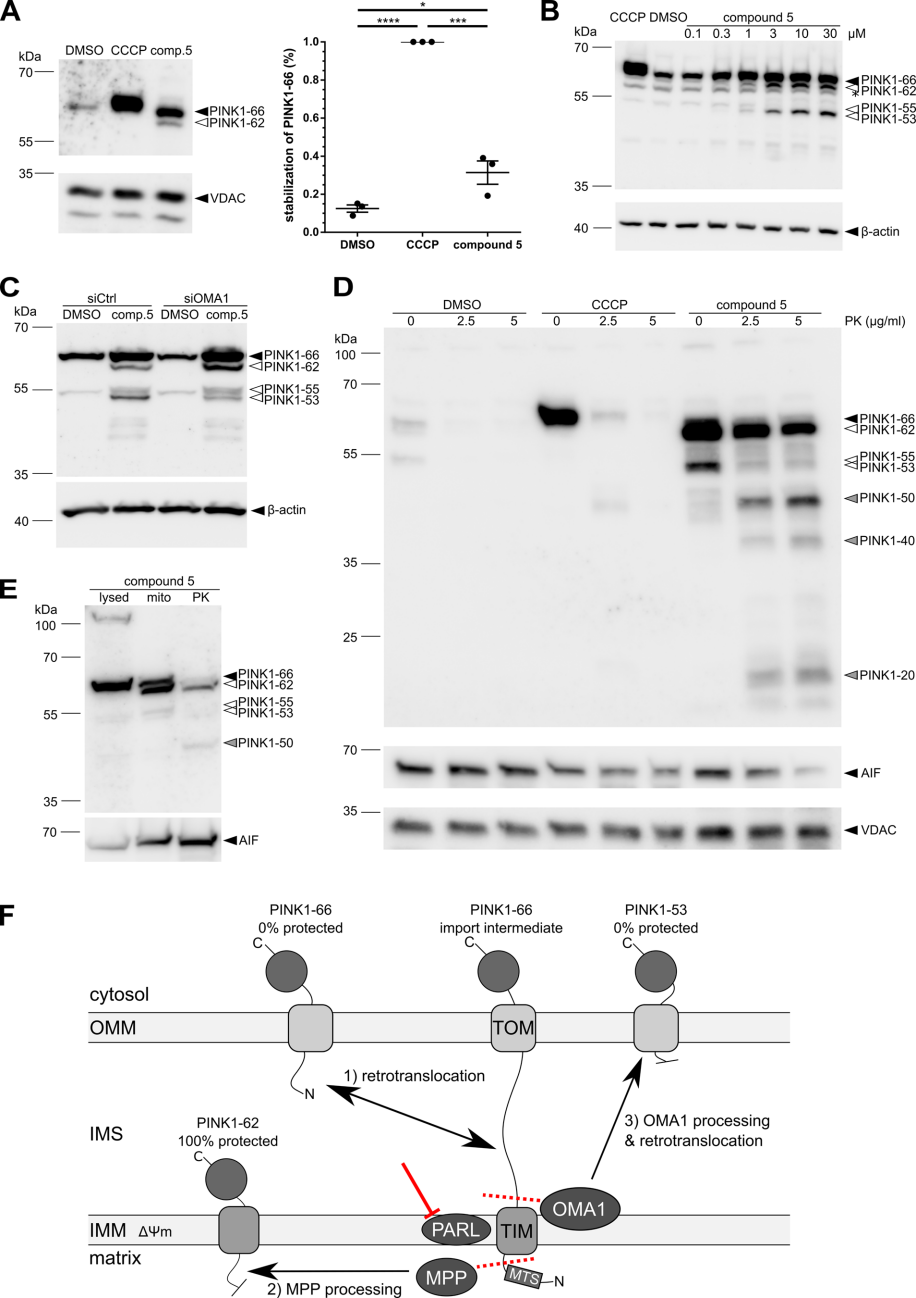
PARL inhibitor stabilizes PINK1 and triggers its alternative cleavage and trafficking in living cells. (A) Compound **5** inhibits PARL-catalyzed PINK1 cleavage under endogenous expression levels. HEK293T cells were treated for 8 h with 5 μM compound **5**, DMSO, or CCCP before harvesting. Harvested cells were subjected to a sodium carbonate fractionation and analyzed via immunoblot. The percentage of PINK1–66 stabilization was quantified compared to PINK1–66 levels in CCCP condition. Levels under compound **5** treatment differ significantly from levels in the DMSO condition (*p ≤ 0.05, ***p ≤ 0.001, ****p ≤ 0.0001, unpaired *t*-test; means ± SEM, *n* = 3). VDAC was used as fractionation control. (B) Compound **5** inhibits the PARL-cleavage of overexpressed PINK1 in a dose-dependent manner. HEK293 T-REx cells overexpressing PINK1 were treated for 8 h with compound **5**, DMSO, or CCCP as indicated before harvesting and analyzed via immunoblot. PINK-66, the PINK1 fragment that has been cleaved by MPP (PINK1–62), and an alternative cleavage fragment (PINK1–53) are all stabilized by compound **5**. No stabilization of PINK1–55 generated by the PARL-catalyzed cleavage is observed. β-actin was used as the loading control. (C) Alternative cleavage of PINK1 (PINK1–53) under compound **5** treatment is reduced under OMA1 knockdown in HEK293 T-REx cells overexpressing PINK1. Cells were treated for 8 h with 10 μM compound **5** or DMSO before harvesting and analyzed via immunoblot. β-actin was used as a loading control. (D) Compound **5**-induced cleaved PINK1 forms are partially protected from proteinase K treatment. HEK293 T-REx cells overexpressing PINK1 were treated for 3 h with 5 μM compound **5**, DMSO, or CCCP before harvesting and subjection to proteinase K (PK) protection assay and analyzed via immunoblot. AIF and VDAC were used as fractionation controls. (E) Compound **5**-stabilized PINK1–66 stays import-competent and can be cleaved by MPP. HEK293 T-REx cells overexpressing PINK1 were treated for 6 h with 5 μM compound **5** before harvesting. Cells were either lysed directly (lysed), subjected to a subcellular fractionation to isolate mitochondria (mito), or subjected to protease protection assay (PK) and analyzed via immunoblot. AIF was used as fractionation control. (F) PINK1 sub-mitochondrial trafficking scheme under PARL inhibitor conditions. Full-length PINK1–66 is imported via the canonical translocase of the outer mitochondrial membrane (TOM) and the translocase of the inner mitochondrial membrane (TIM). Due to prolonged interaction with the cytoplasmic chaperone Hsp90,^51^ PINK1–66 forms an import intermediate spanning the OMM and the IMM. In the presence of PARL inhibitor, the MTS of PINK1–66 can be cleaved by MPP, resulting in a stably inserted IMM PINK1–62 form. Additionally, PINK1–66 import intermediate can be processed by OMA1. The cleavage product (PINK1–53) then associates with the OMM by an unknown mechanism. As an alternative fate, the PINK1–66 import intermediate can directly retrotranslocate, leading to an OMM-anchored species that activates the Parkin-dependent mitophagy pathway.

We first focused on the effect that acute PARL inhibition by compound **5** has on PINK1 processing. By using this PARL inhibitor, we aimed to better dissect the fate of PINK1 and separate this pathway from secondary adaptation effects caused by long-term PARL ablation via knockdown or knockout. Indeed, applying compound **5** shows that PARL inhibition has a comparable effect on PINK1 as does CCCP: Immunoblot analysis of mitochondrial membrane proteins revealed that endogenous PINK1 remains largely uncleaved and is stabilized in its 66 kDa form (PINK1–66, Figure 4A). Compound **5** treatment however also stabilizes an additional PINK1 form with an apparent molecular weight of 62 kDa (PINK1–62, Figure 4A). We conclude that this smaller cleavage fragment corresponds to PINK1 inserted in the IMM that had its matrix-targeting signal (MTS) cleaved off by the mitochondrial processing peptidase (MPP),^49^ a process that is not possible under CCCP treatment due to the block of protein import as a consequence of disruption of the mitochondrial membrane potential. To confirm that compound **5** does not indirectly act on PINK1 by affecting the membrane potential, we employed a JC-1 assay comparing the ratios of JC-1 aggregates (intact mitochondrial membrane potential) to JC-1 monomers (depolarized mitochondrial membrane). We show that while CCCP treatment lowers the membrane potential as evidenced by the lower aggregate to the monomer ratio, compound **5** does not significantly alter the membrane potential as compared to DMSO condition, consistent with a specific effect on PARL (Figure S2). This is another sign that the compound can be used very specifically to investigate PARL and its role in tuning mitophagy without artificial disturbance of the membrane potential. To corroborate this effect, we analyzed the fate of PARL-generated cleavage fragments in Flp-In HEK293 T-REx cells with an inducible PINK1 overexpression, since analysis of the endogenous protein is not very practical due to its low overall expression levels.

Interestingly, blocking PARL-catalyzed PINK1 processing by compound **5** leads to a dose-dependent stabilization of PINK1–66, the MPP-cleaved form PINK1–62, as well as another smaller molecular weight species (PINK1–53, Figure 4B). Knockdown of OMA1, the metalloprotease that is co-regulated with PARL in the SPY complex,^36^ significantly reduced the steady-state level of this alternatively cleaved PINK1–53 form seen under compound **5** treatment, accompanied by a slight increase in the MPP-cleaved species PINK1–62 (Figure 4C). It would thus appear that similar to mistargeting of PINK1,^50^ chemical inhibition of PARL activates OMA1 to cleave a certain portion of PINK1 in the intermembrane space. Taken together with the JC-1 assay, these results indicate that mitochondrial import of PINK1 is unhindered by compound **5**. However, PARL inhibition seems to lead to various different fates of PINK1.

To further illuminate the fate of the differentially processed PINK1 forms, we employed a protease protection assay by treating isolated mitochondria with proteinase K (Figure 4D). Treatment with proteinase K digests all PINK1 present in the mitochondria isolated from vehicle or CCCP-treated cells, whereas in compound **5**-treated mitochondria, most of the MPP-cleaved form PINK1–62 remains protease resistant and is thus localized within the mitochondria. However, we could observe that some amount of PINK1–62 is still digested by proteinase K. As a small fraction of the IMS protein AIF also becomes accessible for proteinase K in the mitochondria treated with CCCP or compound **5**, we suggest that this partial digestion of PINK1–62 does not indicate an additional OMM localization but rather an artificial slight disruption of the OMM, making small fractions of both AIF and PINK1–62 subject to proteinase K. The fractions of PINK1 that remain accessible to proteinase K are cleaved into fragments with the apparent molecular weight of 50, 40, and 20 kDa, respectively (Figure 4D). The low abundance of PINK1–66 here can be explained by the need to perform mitochondrial isolation prior to the proteinase K treatment, resulting in a continuous processing of PINK1–66 by MPP during this timeframe (Figure 4E). The simultaneous presence of a proteinase K sensitive and an MPP-accessible PINK1 suggests that compound **5** treatment results in a heightened quantity of a PINK1 import intermediate. Together with the finding that some PINK1 is localized within the mitochondria, fully protected (PINK1–62), whereas another fraction at the surface remains proteinase K-sensitive (PINK1–66, PINK1–53); this indicates that PARL inhibition by compound **5** reveals alternative PINK1 trafficking pathways (Figure 4F).

### PARL Inhibitor Induces Parkin Recruitment to Mitochondria

**Figure 5:**
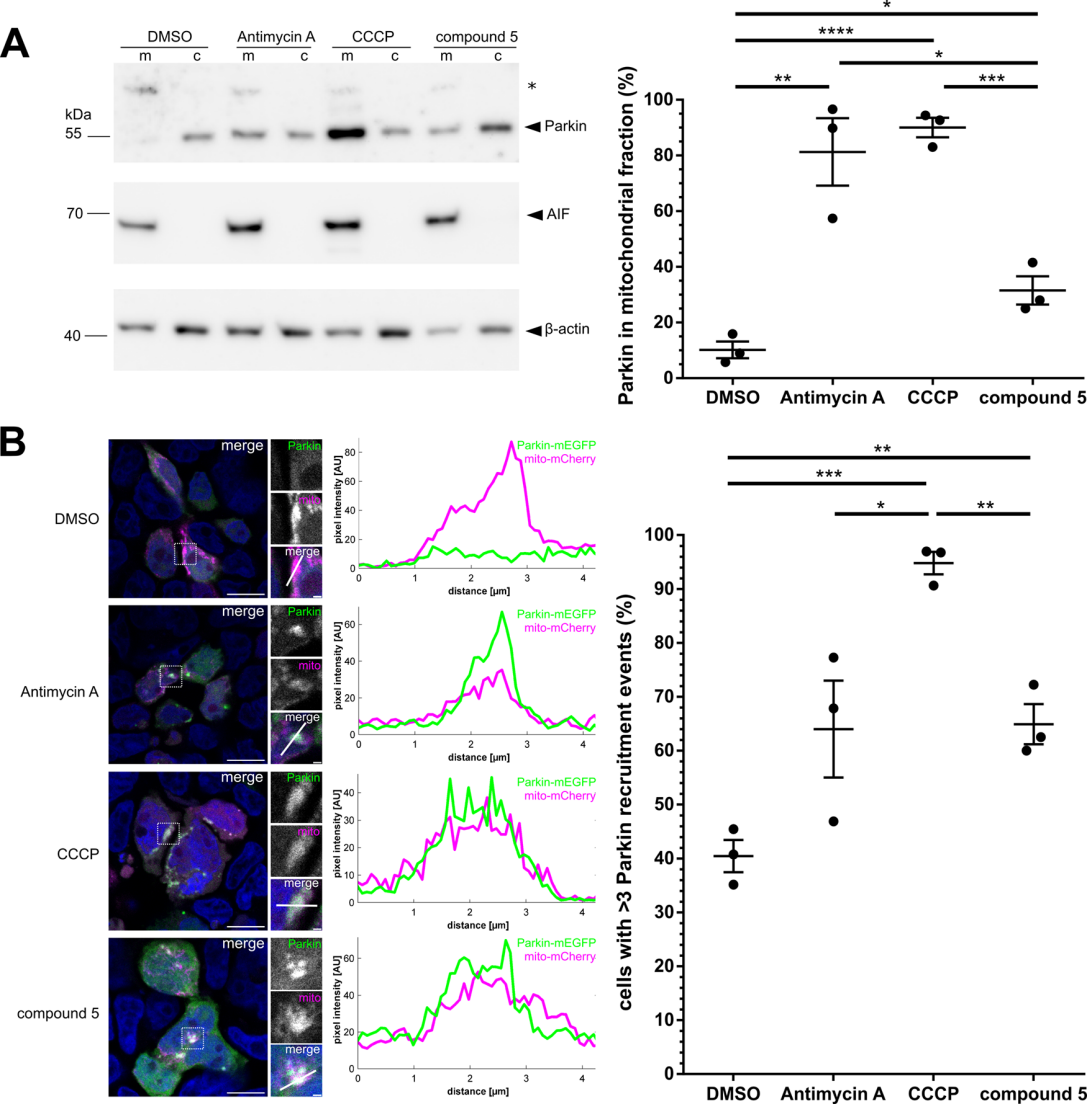
PARL inhibitor affects Parkin recruitment to the mitochondria. (A) HEK293 T-REx cells overexpressing PINK1 were transfected with HA-Parkin-IRES-GFP and treated for 22 h with 5 μM compound **5**, DMSO, CCCP, or Antimycin A. Harvested cells were subjected to a subcellular fractionation and analyzed via immunoblot (m = mitochondrial fraction, c = cytosolic fraction). Parkin levels recruited to the mitochondrial fraction under compound **5** treatment differ significantly from levels recruited under DMSO control conditions (*p ≤ 0.05, **p ≤ 0.01, ***p ≤ 0.001, ****p ≤ 0.0001, unpaired t-test; means ± SEM, *n* = 3). AIF and β-actin were used as fractionation controls. (B) HEK293 T-REx cells overexpressing PINK1 were transfected with mito-mCherry and Parkin-mEGFP and treated for 3 h with 5 μM compound **5**, DMSO, CCCP, or Antimycin A. On the left, representative fluorescence microscopy images of the four conditions are shown, with Hoechst-labeled nuclei in blue (scale bars 10 μm, insets scale bars 1 μm). The middle column shows the corresponding pixel intensity plots for the white line in the merge inset image, demonstrating Parkin recruitment to the mito-mCherry-labeled mitochondria via the overlapping increase of the channel intensity values (Antimycin A, CCCP, compound **5**) or a lack thereof (DMSO). The quantification of Parkin recruitment events to the right indicates significant differences in the recruitment efficiency (*p ≤ 0.05, **p ≤ 0.01, ***p ≤ 0.001, unpaired *t*-test; means ± SEM, *n* = 3, N(DMSO) = 86, N(Antimycin A) = 82, N(CCCP) = 96, N(compound **5**) = 80).

In order to determine to which extent PARL inhibition by compound **5** stabilizes PINK1 at the OMM and activates the PINK1/Parkin mitophagy pathway, we first used subcellular fractionation to test whether compound **5** causes recruitment of Parkin to the mitochondria. Consistent with previous reports, we detected a significant recruitment of Parkin to the mitochondrial fraction in cells treated with CCCP.^52−54^ Likewise, Antimycin A,^55,56^ which blocks the respiratory chain leading to aberrant reactive oxygen species (ROS) production, activates the PINK1/Parkin pathway (Figure 5A). Consistent with our observation of compound **5**-induced stabilization of a PINK1 fraction at the OMM, we detected Parkin recruitment to the mitochondrial fraction, albeit in a lower quantity than in the two chemically induced stress conditions CCCP and Antimycin A (Figure 5A). These lower effect levels indicate that blocking PARL by compound **5** without mitochondrial stress causes a more subtle effect than uncoupling the inner membrane potential.

To investigate the Parkin recruitment more directly without previous mitochondrial isolation, we next used a microscopy-based approach. We observed significant recruitment of mEGFP-tagged Parkin to the mitochondria in compound **5**-treated cells compared to DMSO or CCCP (Figure 5B), strengthening the findings from Figure 5A. As in the subcellular fractionation approach, the Antimycin A treatment appears to have a high variance between individual experiments. As opposed to the fractionation approach, the Parkin recruitment under Antimycin A treatment in the microscopy approach is not significantly different from Parkin recruitment under DMSO conditions. It thus seems that Antimycin A is, in these experiments, not a very robust and consistent mitochondrial stressor. Interestingly, the morphology of the mitochondria under both Antimycin A and compound **5** treatment differed significantly from the CCCP-treated cells, which showed severe rounding and clustering. Taken together, this indicates that compound **5** has key advantages over other commonly used chemicals for PARL/PINK1/Parkin investigations, such as CCCP or Antimycin A: Compound **5** leads to a robust, specific activation of the PINK1/Parkin pathway without major secondary effects on mitochondrial properties.

## Conclusions

This report details the development of a generalizable in vitro assay and of potent ketoamide inhibitors of human mitochondrial rhomboid protease PARL. This generally showcases the power of ketoamide inhibitors to interfere with rhomboid-catalyzed intramembrane proteolysis. Since rhomboid proteases are still scarcely characterized, the development of specific inhibitors is a key strategic advance in characterizing their molecular function. Importantly, this study discloses a way of pharmacologically blocking PARL to boost PINK1/Parkin-dependent mitophagy, whose enhancement is explored as a therapeutic approach for the treatment of Parkinson’s disease.

## Experimental Section

### General Biochemicals

Lipids were purchased from Avanti Polar Lipids, and buffer components and other chemicals were from Sigma unless specified otherwise. DIBMA was a kind gift of Sandro Keller (University of Gratz, Austria).

### Chemical Synthesis

Chemical synthesis of substrates and inhibitors is described below. Analytical characterization data by mass spectrometry and NMR of compounds **1**–**7** are listed in the Supporting Information file. All compounds were >95% pure by HPLC analysis.

#### Synthesis of 7-Amino-4-methylcoumarin Labeled Substrates (Compounds **1**, **3**, and **4**)

##### Synthesis of the Peptide Part

The peptide part was synthesized on solid support, using 2-chlorotrityl resin (Substitution 1.6 mmol/g). The first amino acid (Fmoc-protected) was coupled to the resin in a quantity of 1 molar equivalents (equiv) in the presence of 4 equiv DIEA, dissolved in 6–7 mL of dry DCM by shaking at room temperature. The resin was then washed with DCM/MeOH/DIEA = 17:2:1 (3 × 3–4 mL), DCM (3 × 3 mL), DMF (2 × 3 mL), and DCM (5 × 3 mL). After drying in vacuum, the resin was subjected to amino acid analysis. The amino acids were coupled in a quantity of 4 equiv, in the presence of 5 equiv HOBt and 7 equiv DIC, dissolved in 2–3 mL of DMF, using the Fmoc-chemistry protocol and using 20% piperidine in DMF (20 min) for Fmoc removal. N-terminal acetylation was performed using acetic anhydride/DIEA (10 equiv/10 equiv), dissolved in a minimal volume of DMF. The cleavage of the peptide off the resin was performed by treating the dry peptidyl resin with DCM/ TFE/AcOH = 7:2:1 (6–7 mL) and shaking for 1 h, followed by adding further 2–3 mL of the same solution and 10 min incubation. The resin was then washed twice with 3 mL of DCM/TFE (4:1). The combined filtrates of cleavage and washings were vacuum-evaporated and freeze-dried.

##### Synthesis of the BOC-Ala-4-methylcoumarin Fragment

BOC-Ala-OH (250 mg, 1.32 mmol) and 7-amino-4-methylcoumarin (233 mg, 1.33 mmol) were dissolved in 2.3 mL of dry DMF. Then, the solution was cooled to 0 °C, and DCC (308 mg, 1.43 mmol), dissolved in 0.7 mL of dry DMF, was added dropwise over 1 h. The mixture was stirred for 2 h at 0 °C under inert conditions and then for 24 h at ambient temperature (about 25 °C). Dicyclohexyl urea was removed by filtration and the solution was evaporated to dryness. The oil-like residue was triturated with 10% KHSO_4_. After the filtration of the solution, the residue was washed with 0.5 M NaHCO_3_ (in small amounts) and water until neutral pH was reached. The product was isolated by RP HPLC, using a C_18_ column and gradient of 15–50% actetonitrile:0.1% (v/v) TFA in water.

Yield: 150 mg (33%). 1H NMR (400 MHz, DMSO) δ (ppm) = 10.40 (s, 1H, CONH), 7.78 (d, *J* = 2.1 Hz, 1H, aromatic), 7.73 (d, *J* = 8.7 Hz, 1H, aromatic), 7.50 (d, *J* = 8.5 Hz, 1H, aromatic), 7.20 (d, *J* = 7.3 Hz, 1H, CONH), 6.27 (s, 1H, CH), 4.13 (t, *J* = 7.2 Hz, 1H, CH), 2.40 (s, 3H, CH_3_), 1.39 (s, 9H, BOC), 1.28 (d, *J* = 7.1 Hz, 3H, CH_3_).

13C NMR (101 MHz, DMSO) δ (ppm) = 173.19, 160.49, 155.73, 154.12, 153.58, 142.88, 126.41, 115.65, 115.43, 112.72, 106.06, 78.60, 51.10, 28.67, 18.45.

MS: Calculated monoisotopic mass for C_18_H_22_N_2_O_5_ 346.15, found [M + H^+^] C_18_H_23_N_2_O_5_ = 347.39.

##### Deprotection and Coupling of BOC-Ala-4-methylcoumarin to the Peptide

Deprotection of BOC-Ala-4-methylcoumarin was performed by dissolving it in 1 mL of TFA and subsequent sonication. The TFA was removed by flushing with nitrogen. The peptides were dissolved in dry DMF, and then DIEA (1.8 equiv) was added, followed by TSTU (1.8 equiv). The mixture was stirred overnight under inert conditions, and the reaction course was monitored by HPLC analysis. Then, the solution of H-Ala-4-MC.TFA (1 equiv) in 0.5 mL of DMF, alkalized with DIEA to pH 8, was added to the solution of the activated peptide. The reaction mixture was stirred overnight at pH 8, and the reaction course was monitored by HPLC analysis. Then, DMF was evaporated and the product was triturated with 10% KHSO_4_, followed by washing with 0.5 M NaHCO_3_ (5–10 mL) and water until neutral pH. The crude product was dried in vacuum.

##### Deprotection of the Orthogonal Protecting Groups and Purification

In case the peptides contained orthogonal protection groups (f.e. Arg(Pbf)), those were removed by the treatment of the peptidyl-4mc with the mixture TFA/TIS/water = 95:2.5:2.5 (1–1.2 mL) at room temperature for 1–1.5 h. Then, TFA was removed by flushing with nitrogen and the crude peptide was precipitated with ether, followed by filtering and washing with small amounts of ether. The peptidyl-4mc substrates were purified by RP HPLC (C_18_), using 0.1% (v/v) TFA in the water/acetonitrile gradient.

#### Synthesis of Peptidyl-α-Ketoamide Inhibitors (Compounds **2**, **5**, **6**, **7**)

The peptide part was synthesized as described in the section “Synthesis of the Peptide Part”. The precursor of the warhead BOC-Ala-CH(*OH*)CO-NH-(CH_2_)_4_-Ph/BOC-Phe-CH(*OH*)CO-NH-(CH_2_)_4_-Ph was synthesized as previously described,^17,19,57^ followed by Dess–Martin oxidation^17,19^ to yield the final N-substituted peptidyl-α-ketoamides.

The BOC removal was performed by sonicating 0.7 mL of solution of the warhead in acetonitrile, containing 1.3 equiv pTSA·H_2_O until white precipitate was formed, followed by the evaporation of acetonitrile.^19^ The coupling to the peptide was done by the activation of the peptide with PyBrOP (1.5 equiv), HOBt (1 equiv), and DIEA (3 equiv).^58^ Whenever the peptidyl-α-ketoamide contained orthogonal protecting groups, the deprotection procedure was performed as described above. The final product was purified by RP HPLC (C_18_), using the water +0.1% TFA/acetonitrile gradient.^17,19^

#### EDANS/Dabcyl-Labeled Peptides

The major part of the peptide synthesis was performed on a solid support Tenta Gel S Rinkamide resin (substitution 0.24 mmol/g) on a PS3 peptide synthesizer (Protein technologies, USA), using the Fmoc-chemistry protocol in a scale of 0.1 mmol. The coupling of the Fmoc-Glu(EDANS)-OH and Fmoc-Lys(Dabcyl)-OH was done manually in the same scale in a fourfold excess, using HBTU (4 equiv)/HOBt (4 equiv)/DIEA (6 equiv) in 3–4 mL of DMF. Then, the peptide was cleaved from the resin by the mixture of trifluoroacetic acid/triisopropylsilane/water = 95:2.5:2.5 for 3 h. The mixture was concentrated under nitrogen, and the crude peptide was precipitated with cold ether. The precipitate was washed by ether, dried, and purified by RP HPLC (gradient from 30 to 80% B).

#### HPLC Analysis and Purification

The chromatographic conditions were Module Jasco PU 1580 Series (Jasco, Japan) with a preparative RP C_18_ column (250 × 20 mm, 10 μm particle size, Watrex International, Inc., San Francisco, California, USA), using water +0.1% trifluoroacetic acid (A)/acetonitrile (B) as mobile phases, at 10 mL/min flow rate over 60 min at room temperature. The elution was monitored by absorption at 210 nm, using a UV–vis 1575 detector. The fractions corresponding to the major peak (the desired product) were combined, frozen, and lyophilized, giving the pure peptide. Analytical runs were done on the same module using the Watrex C_18_, 250 × 4.6 mm, 5 μm particle size column (Watrex International, Inc., San Francisco, California, USA) at 1 mL/min flow rate using various solvent gradients ranging from solvent A to solvent B as defined above over 36 min at room temperature.

#### Liquid Chromatography–Mass Spectroscopy

Agilent Technologies liquid chromatograph was coupled with a TOF 6230 ESI-MS detector. Gradient: 2%B to 100%B over 10 min on a 1.7 μm particle size C_18_, 100 × 2.1 mm RP HPLC column (Waters) at a flow rate of 0.3 mL/min, where solvent A is water with 0.1% formic acid (FA) and solvent B is acetonitrile with 0.1% FA.

#### NMR Spectroscopy

NMR spectra were acquired on a Bruker AV 400 MHz at room temperature.

### Antibodies

The primary and secondary antibodies used throughout the study are listed in Table 1.

**Table 1:** 

reagent	WB dilution	source	identifier
anti-AIF mouse mAb	1:500	Santa Cruz Biotechnology	#sc-13116
anti-β-actin mouse mAb	1:2000	Sigma-Aldrich	#A1978
anti-FLAG M2 mouse mAb	1:1000–2000	Sigma-Aldrich	#F1804
anti-HA.11 mouse mAb	1:1000	BioLegend	#901502
anti-Myc-Tag (71D10) rabbit mAb	1:1000	Cell Signaling Technology	#2278
anti-penta-His mouse mAb	1:2000	Invitrogen	#P21315
anti-PINK1 rabbit pAb	1:1000	Novus biologicals	#BC100-494
anti-actin mouse mAb, clone C4	1:1000	Chemicon	#MAB1501R
anti-VDAC rabbit pAb	1:1000	Invitrogen	#PA1-954A
anti-mouse IgG, DyLight 680	1:10,000	Invitrogen	#SA5-10170
anti-mouse IgG, DyLight 800	1:10,000	Invitrogen	#SA5-10172
anti-rabbit IgG, DyLight 800	1:10,000	Invitrogen	#SA5-10044
anti-mouse IgG (H + L) donkey-HRP	1:20,000	Dianova	#715-035-150
anti-rabbit IgG (H + L) donkey-HRP	1:20,000	Dianova	#711-035-152

### DNA Constructs, Cloning, and RNA Interference

For in vitro translation of PARL, mature human PARL cDNA (encoding amino acids 53-end) was cloned into the pIVEX2.3d plasmid using *Nde*I and *Xho*I restriction enzymes. For plasmid pcDNA3.1_hPGAM5-Myc, the DNA encoding human PGAM5 was amplified from pcDNA3.1_C_(K)-hPGAM5-DYK (GenScript), extended by a Myc-tag at the C-terminus, and cloned into pcDNA3.1 (Invitrogen) using Gibson Assembly.^59^ All constructs were sequenced. The following plasmid constructs have been described previously: pcDNA3.1/PGAM5-FLAG^29^ pCDH/HA-Parkin-IRES-GFP,^51^ pEGFP-C1/Parkin-mEGFP,^33^ and mito-mCherry.^60^ Small interfering RNA (siRNA)-oligonucleotides OMA1 #776 (4392420, ID s41776) and nontargeting control siRNA (4390843) were purchased from Ambion.

### GlpG Expression and Purification

Full-length GlpG with C-terminal His-tag was overexpressed from pET25b + in *E. coli* C43(DE3)^61^ in the LB medium. Transformed bacteria were grown at 37 °C until OD_600_ reached 0.6 and then were induced with 0.4 mM IPTG and shaken at 16 °C for 20 h. Cells were harvested by centrifugation at 6000 × *g* at 4 °C for 20 min, and cell pellets were resuspended in PBS. Cells were lysed using a CF1 cell disruptor (Constant Systems) in four disruption cycles at 27, 30, 33, and 35 kPsi. Cell debris was removed by centrifugation at 10,000 × *g* at 4 °C for 30 min, and membranes were harvested by ultracentrifugation at 100,000 × *g* at 4 °C for 2 h. Protein concentration in membranes was determined using Pierce 660 nm Protein Assay Reagent (Thermo Scientific).

Membranes were solubilized at a membrane protein concentration of 6 mg/mL in 25 mM HEPES (pH 7.4), 300 mM NaCl using 2.5% (w/v) DIBMA at room temperature,^25^ or 1.5% (w/v) DDM (GLYCON Biochemicals) at 4 °C overnight. Unsolubilized membranes were removed by ultracentrifugation at 100,000 × *g*, 4 °C for 45 min and solubilized proteins were bound to 2 mL of Ni-NTA agarose (Qiagen) per liter of bacterial culture at 4 °C overnight. The resin was washed with 10 column volumes of 25 mM HEPES (pH 7.4), 300 mM NaCl, 20 mM imidazole (with 0.1% (w/v) DDM for DDM sample), and 15 column volumes of 25 mM HEPES (pH 7.4), 300 mM NaCl, and 30 mM imidazole (with 0.1% (w/v) DDM for the DDM sample). GlpG was eluted in 4 column volumes by 25 mM HEPES (pH 7.4), 300 mM NaCl, and 250 mM imidazole (with 0.1% (w/v) DDM for DDM sample). Imidazole was removed by dialysis of the elution fraction against 25 mM HEPES (pH 7.4) and 150 mM NaCl (with 10% (v/v) glycerol, 0.1% (w/v) DDM for the DDM sample) using a dialysis membrane with 12–14 kDa cut-off (Spectrum Labs). The protein concentration of the DDM solubilized sample was determined by absorption at 280 nm, and the protein concentration of the DIBMA-solubilized sample was determined based on band intensities on a Coomassie-stained polyacrylamide gel by ImageJ using the DDM-solubilized sample as the reference.

### Mass Spectrometry

For LC–MS of compound **1** and its cleavage, 1 mM compound was incubated with 13 μM GlpG WT or S201T, 1 μM elastase or buffer in in 50 mM potassium phosphate pH 7.4, 150 mM NaCl, 20% (w/v) glycerol, 0.05% (w/v) PEG8000, 0.05% (w/v) DDM, 10% (v/v) DMSO at 37 °C for 1 h. Samples were diluted 5-fold with buffer, and enzymes were removed by centrifugation through a protein concentrator with a 10 kDa cut-off (Sartorius) prior to LC–MS. Data were evaluated using GraphPad Prism 9 (GraphPad Software, Inc.). Data shown are representative of two independent experiments.

### Emission Spectra of Compound **1** Cleavage

Emission spectra of aminomethyl coumarin and cleavage of compound **1** were monitored in 50 mM potassium phosphate pH 7.4, 150 mM NaCl, 20% (w/v) glycerol, 0.05% (w/v) PEG8000, 0.05% (w/v) DDM, 10% (w/v) DMSO. The compounds (1 mM) were incubated with 10 μM GlpG WT or S201T, 1 μM elastase, or buffer, respectively, as indicated in the legend of Figure 1A at 37 °C for 1 h. Samples were diluted 8-fold with buffer, and emission spectra were measured in 96-well black HTS plates (Greiner Bio-One) by a plate reader (Tecan Infinite M1000) using an excitation wavelength of 355 nm. Data were evaluated using Excel (Microsoft) and GraphPad Prism 9 (GraphPad Software, Inc.). The data shown are representative of three independent experiments.

### Activity and Inhibition Assay of GlpG

Activity and inhibition assays of GlpG shown in Figure 1B–D were carried out in 50 mM potassium phosphate pH 7.4, 150 mM NaCl, 20% (v/v) glycerol, 0.05% (w/v) PEG8000, 0.05% (w/v) DDM, and 5% (v/v) DMSO. For activity measurements with DIBMA-solubilized samples in Figure 1E, 25 mM HEPES (pH 7.4), 150 mM NaCl, 10% (w/v) glycerol, and 5% (v/v) DMSO (with 0.1% (w/v) DDM for the DDM-solubilized active and inactive control were used. GlpG was used at 100 nM final concentration for inhibition assays and at 400 nM concentration for all other activity assays if not stated otherwise. Substrate and inhibitor concentrations were determined by quantitative amino acid analysis. If not indicated otherwise, compound **1** was used at 100 μM and the transmembrane substrate KSp96^17^ at 25 μM final concentration. For inhibition assays, GlpG was incubated with different concentrations of compound **2** at 37 °C for 1 h prior to adding the substrate compound **1**. All activity measurements were performed at 37 °C in 96-well black HTS plates (Greiner Bio-One) and fluorescence was monitored in a plate reader (Tecan Infinite M1000) with excitation and emission wavelengths set to 355 and 450 nm, respectively, for compound **1**, and 335 and 493 nm, respectively, for the transmembrane substrate KSp96. Data were evaluated using Excel (Microsoft) and GraphPad Prism 9 (GraphPad Software, Inc.).

### GlpG Activity Dependence on Detergent Concentration

Activity measurements of GlpG with varying DDM concentrations were obtained in 20 mM HEPES (pH 7.4), 150 mM NaCl using 100 nM GlpG, and DDM at the concentration indicated in the graphs. Buffer for measurements with compound **1** contained additionally 5% (v/v) DMSO. Compound **1** and transmembrane substrate were used at 10 μM final concentration. Activity measurements were performed at 37 °C in 96-well black HTS plates (Greiner Bio-One), and fluorescence was monitored in a plate reader (Tecan Infinite M1000) with excitation and emission wavelengths set to 355 and 450 nm for compound **1** and 335 and 493 nm for the transmembrane substrate, respectively. Data were evaluated using Excel (Microsoft) and GraphPad Prism 9 (GraphPad Software, Inc.).

### In Vitro Translation of PARL into Liposomes

Liposome preparation and cell-free protein expression of PARL were carried out essentially as described previously.^27^ Liposomes resembling the lipid composition of the IMM were prepared by mixing 40% DOPC (1,2-dioleoyl-sn-glycero-3-phosphocholine), 33.8% DOPE (1,2-dioleoyl-sn-glycero-3-phosphoethanolamine), 18% CL(18:1)4 (1′,3′-bis[1,2-dioleyl-sn-glycero-3-phospho]-glycerol), 3% DOPS (1,2-dioleoyl-sn-glycero-3-phospho-l-serine), 5% soy-PI (L-α-phosphatidylinositol), and 0.2% rhodamine-PE (1,2-dioleoyl-sn-glycero-3-phosphoethanolamine-N-(lissamine rhodamine B sulfonyl)) in chloroform, followed by the evaporation of chloroform by a stream of nitrogen gas and drying in a vacuum chamber (Binder) for 1 h. Dried lipids were dissolved in buffer (5 mM Tris/HCl, pH 8.5, 10 mM KOAc) and extruded 21 times through a mini extruder (Avanti Polar Lipids) using a polycarbonate membrane with a pore size of 0.1 μM. Wild-type mature PARL (starting at amino acid 53) and its inactive S277A mutant were expressed with a C-terminal hexahistidine tag from a pIVEX2.3d plasmid in a continuous-exchange cell-free expression system^62^ into 6.5 mM liposomes at 30 °C overnight in bacterial lysates with T7 polymerase (CUBE biotech) with 100 mM HEPES (pH 7.4), amino acids (0.55 mM of L-asparagine, alanine, glutamine, glycine, histidine, isoleucine, leucine, phenylalanine, proline, lysine, serine, threonine, valine, and tyrosine and 1.55 mM of L-arginine, cysteine, tryptophan, methionine, aspartate, and glutamate), NTPs (4.8 mm ATP, 3.2 mM CTP, 3.2 mM UTP, and 3.2 mM GTP), 20 mM lithium potassium acetyl phosphate, 20 mM phosphoenolpyruvic acid monopotassium salt, 0.1 mg/mL folinic acid, 0.8 mM EDTA, 20 mM magnesium acetate, 270 mM potassium acetate, 2% PEG8000, 2 mM dithiothreitol, 0.05% NaN_3_, 0.3 U/μL RiboLock R1 RNAse inhibitor (Thermo Scientific), 0.5 mg/mL tRNA *E. coli* MRE600 (Roche), 0.04 mg/mL pyruvate kinase (Roche), and 0.015 mg/mL plasmid DNA. The PARL-containing liposomes were purified by a step-wise sucrose step-gradient centrifugation (0, 15, 30, and 40% sucrose in 5 mM HEPES pH 7.5 with 25 mM NaCl) for 2 h at 200,000 × *g*. Soluble proteoliposomes were harvested at the 0%/15% sucrose interface after the centrifugation. Concentration of PARL was determined by absorption at 280 nm.

### In Vitro Assay for PARL Activity

The PARL activity assay in vitro was carried out in 5 mM Tris, 5 mM Mg(OAc)_2_, 25 μM Zn(OAc)_2_, 0.1 μg/μL BSA, 30% DMSO, pH 8.0 using PARL in liposomes at 0.05 mg/mL. Compound **3** and **4** were used at 5 μM final concentration based on quantitative amino acid analysis. Both compounds in DMSO were dissolved into the reaction buffer by vigorous mixing at 1000 rpm, 37 °C for 40 min prior to the addition of PARL proteoliposomes. The cleavage reaction was carried out at 37 °C in 96-well black HTS plates (Greiner Bio-One) in a plate reader (Tecan Infinite M1000) with excitation and emission wavelengths set to 355 and 450 nm, respectively. Data were evaluated using Excel (Microsoft) and GraphPad Prism 9 (GraphPad Software, Inc.).

PARL inhibition in vitro was measured in 5 mM Tris, 5 mM Mg(OAc)_2_, 25 μM Zn(OAc)_2_, 0.1 μg/μL BSA, 10% DMSO, pH 8.0 using PARL in liposomes at 0.07 mg/mL and compound **4** at 100 μM final concentration. Inhibitor concentrations were determined by quantitative amino acid analysis. Inhibitors in DMSO were dissolved at the respective concentrations into the reaction buffer by vigorous mixing at 1000 rpm, 37 °C for 40 min and then pre-incubated with PARL in liposomes at 37° for 1 h. The cleavage reaction was started by adding compound **4** and carried out at 37 °C in 96-well black HTS plates (Greiner Bio-One). Fluorescence was read in a plate reader (Tecan Infinite M1000) with excitation and emission wavelengths set to 355 and 450 nm, respectively. Data were evaluated using Excel (Microsoft) and GraphPad Prism 9 (GraphPad Software, Inc.). The data shown are representative of at least three independent experiments.

### Cell Lines and Transfection

PARL inhibition assays in cells were carried out in Flp-In HEK293 T-REx PARL knockout cells stably transfected with tetracycline inducible PARL-FLAG grown in DMEM (Gibco) supplemented with 10% (v/v) tetracycline-free fetal bovine serum (Clontech). For the inhibition assay, 0.7 × 10^6^ cells were seeded per well of a 6-well plate, and the next day transfected with 5 μg of human PGAM5-Myc in pcDNA and 15 μg of branched polyethylenimine (Polysciences) per well. Four hours after transfection, the PARL expression was induced by adding tetracycline to 1 μg/mL. Eight hours after transfection, the inhibitors were added to each well in different concentrations in serum-free DMEM with 1% DMSO.

Inducible stable Flp-In HEK293 T-REx cells expressing PINK1 were described previously.^33^ HEK293 T-REx PINK1 and HEK293T cells were grown in DMEM (Gibco) supplemented with 10% (v/v) fetal bovine serum (ThermoFisher), 1% (v/v) GlutaMAX (Gibco), 1% (v/v) sodium pyruvate (Gibco), 125 μg/mL hygromycin (Invitrogen), and 10 μg/mL blasticidin (Gibco) or DMEM supplemented with 10% (v/v) fetal bovine serum, respectively, at 37 °C in 5% (v/v) CO_2_. For microscopy, cells were seeded on poly-l-lysine (Sigma)-coated slides. Transient plasmid transfections were performed using 25 kDa linear polyethylenimine (Polysciences). Plasmid (100 ng) encoding HA-Parkin-IRES-GFP, mito-mCherry or Parkin-mEGFP, or 500 ng plasmid encoding PGAM5-FLAG were used per 6-well. Total transfected DNA was 2 μg/well. For siRNA transfection, 2 × 10^5^ cells were seeded per 6-well and on the next day transfected with 20 nM siRNA-oligonucleotide using lipofectamine RNAiMAX (ThermoFisher). Cells were analyzed 27 h post-transfection or five days post-siRNA-transfection. HEK293 T-REx PINK1 cells were induced with 0.3 μg/mL doxycycline for 24 h before further processing. Hoechst staining (1 μg/mL) was done immediately before adding treatment to the cells. Treatments were administered for the indicated times before processing; CCCP (Sigma) was always used as 10 μM for 3 h and Antimycin A (Sigma) as 30 μM for 3 h.

### Fractionation and Protease Protection Assay

Unless mentioned otherwise, all steps were performed at 4 °C. For subcellular fractionation to separate cellular content into cytosolic and mitochondrial fractions, cells were washed with PBS, harvested in PBS-EDTA (1 mM EDTA, 0.2 g/L d-glucose), and centrifuged at 500 × *g* for 5 min. The pellet was reconstituted in isolation buffer (250 mM D-sucrose, 10 mM Tris pH 7.4, 10 mM HEPES, 0.05 mM EGTA) plus an EDTA-free complete protease inhibitor cocktail (PI, Roche) and incubated for 10 min. Cells were lysed by passing through a 27-gauge needle six times and then centrifuged at 200 × *g* for 5 min. The supernatant was centrifuged at 10,000 × *g* for 10 min. The resulting supernatant (cytosolic fraction) was subjected to a 10% trichloracetic acid precipitation, then was washed with acetone at room temperature, and was finally resuspended in Tris-glycine SDS-PAGE sample buffer (50 mM Tris-Cl pH 6.8, 10 mM EDTA, 4% glycerol, 2% SDS, 0.01% bromophenol blue, 5% β-mercaptoethanol). The resulting pellet (mitochondrial fraction) was washed in isolation buffer plus PI. The suspension was centrifuged at 10,000 × *g* for 10 min, and the supernatant was discarded. This step was repeated, and then the pellet was resuspended in Tris-glycine SDS-PAGE sample buffer. All samples were heated for 15 min at 65 °C.

For sodium carbonate fractionation to detect endogenous PINK1 signals at enriched mitochondrial membranes, cells were washed and harvested with PBS and centrifuged at 500 × *g* for 5 min. The pellet was reconstituted in hypotonic buffer (10 mM HEPES-KOH pH 7.4, 1.5 mM MgCl_2_, 10 mM KOAc, 0.5 mM DTT, PMSF) plus PI and incubated for 10 min. The suspension was centrifuged at 500 × *g* for 10 min and the pellet was resuspended in hypotonic buffer. Cells were lysed by passing through a 27-gauge needle six times and then centrifuged at 1000 × *g* for 10 min. The supernatant was centrifuged at 100,000 × *g* for 15 min. The resulting pellet (membrane fraction) was resuspended in hypotonic buffer, and then an equal amount of 200 mM Na_2_CO_3_ was added and resuspended. The suspension was incubated for 30 min and then overlaid on a sucrose cushion (100 mM Na_2_CO_3_, 250 mM D-sucrose) and centrifuged at 130,000 × *g* for 15 min. The pellet was resuspended in Tris-glycine SDS-PAGE sample buffer and heated for 15 min at 65 °C.

For protease protection assay, cells were washed with PBS, harvested in PBS-EDTA and centrifuged at 500 × *g* for 5 min. The pellet was reconstituted in EGTA-free isolation buffer plus PI and incubated for 10 min. Cells were lysed by passing through a 27-gauge needle six times and then centrifuged at 200 × *g* for 5 min. The supernatant was centrifuged at 10,000 × *g* for 10 min. The resulting pellet (mitochondrial fraction) was resuspended in EGTA-free isolation buffer, and then proteinase K was added and the suspension was incubated for 30 min (Figure 4E) or 1 h (Figure 4D). Serine protease inhibitor PMSF (2.5 mM) was added and incubated for 15 min. Samples we mixed with 4× Tris-glycine SDS-PAGE sample buffer and heated for 15 min at 65 °C.

### Immunoblotting

For immunoblot analysis of the effects of PARL inhibitors on PGAM5 cleavage, after overnight incubation with inhibitors, cells from each well were harvested in 1× SDS sample buffer containing 20 mM MgCl_2_ and Pierce universal nuclease (250 U/mL, Thermo Scientific) and heated to 65 °C for 10 min. Loading volumes for SDS-PAGE were adjusted by measuring total protein concentration in the lysates with Pierce 660 nm Protein Assay Reagent and Ionic Detergent Compatibility Reagent for Pierce 660 nm Protein Assay Reagent (Thermo Scientific). Samples were resolved by SDS-PAGE using a 12% polyacrylamide gel (Biorad) and electrotransferred onto an Immobilon PVDF membrane (Merck Millipore). Blots were blocked using casein blocker (Thermo Scientific) and hPGAM5 was labeled by rabbit anti-Myc antibody (Cell Signaling Technology, cat. #2278, dilution 1:1000) at 4 °C overnight. Blots were washed three times with TBS-T and incubated with IRDye 800 CW donkey anti-rabbit antibody (Invitrogen, cat. #SA5-10044, dilution 1:10,000) at room temperature for 2 h. Membranes were then again washed three times with TBS-T and one time with TBS. Fluorescence emission at 700 and 800 nM of dried membranes was scanned on Odyssey CLx (LI-COR). Cleavage efficiency and inhibition were analyzed as described^23^ using Image Studio Lite (LI-COR), Excel (Microsoft) and GraphPad Prism 9 (GraphPad Software, Inc.).

For immunoblot analysis in other cases, cells were either processed as described above or washed with PBS and directly lysed with Tris-glycine SDS-PAGE sample buffer and heated for 15 min at 65 °C. Proteins were resolved by Tris-glycine SDS-PAGE, blotted onto an Immobilon PVDF membrane via a semi-dry blotting system, blocked with 5% milk in TBS-T, and incubated with primary antibody in milk solution overnight at 4 °C. Membranes were then washed with TBS-T and incubated with the fitting secondary HRP-coupled antibody. Membranes were washed again before imaging via enhanced chemiluminescence (WesternBright ECL, Advansta). For stripping and reprobing, membranes were either incubated in harsh stripping buffer (62.5 mM Tris pH 7.4, 2% SDS, 0.7% β-mercaptoethanol) at 50 °C for 30 min or in mild glycine stripping buffer (100 mM glycine, 30 mM MgAc, 50 mM KCl, 1% Tween-20, 0.1% SDS, pH 2.2) for 30 min at room temperature. Stripped membranes were washed in TBS-T before continuing with the blocking step and antibody incubation as before. For detection, the ImageQuant LAS 4000 (GE Healthcare) or Amersham ImageQuant 800 (Cytiva) was used. For quantification, ImageJ was used (http://rsb.info.nih.gov/ij/). Immunoblots shown are representative of at least three independent experiments and were evaluated using Excel and GraphPad Prism 9 (GraphPad Software, Inc.).

### JC-1 Assay

Staining of cells for the determination of mitochondrial membrane potential was carried out with the JC-1 mitochondrial staining kit (Sigma-Aldrich) according to the kit’s instructions; stained cells were immediately taken to live cell imaging.

### Microscopy and Image Processing

For fixation, cells were incubated for 15 min with 4% formaldehyde (16% formaldehyde diluted in PBS, Thermo Scientific) and washed in PBS. The cover glasses were mounted with Fluoromount-G (Southern Biotech) on microscope slides. All microscopy was performed on an LSM 780 system (Carl Zeiss) with a Plan-APOCHROMAT 63× 1.4NA oil objective (Carl Zeiss) and pinhole settings of 1 AU with the Zeiss ZEN 2010 software. Image processing and analysis was performed using ImageJ (http://rsb.info.nih.gov/ij/). Parkin recruitment event analysis was carried out in a blinded fashion using the ImageJ plug-in “Blind Analysis Tools” (Jaiswal & Lorenz, https://imagej.net/plugins/blind-analysis-tools). Parkin recruitment events to the mitochondria were counted by hand throughout each z-stack and cell. The percentage of cells that displayed more than three recruitment events was calculated. JC-1 images were handled as summed intensity z-stack projections, as well as subjected to flatfield correction and subtraction of background before measuring the fluorescence levels normalized to the number of cell nuclei. The fluorescence intensity ratio between the red and green signal was calculated and compared to DMSO as a ratio of 1. Data were evaluated using Excel and GraphPad Prism 6.

## Supporting Information Available

Mass spectrometric characterization of compound 1 cleavage by GlpG; mitochondrial membrane potential is not affected by compound 5; and compound characterization data (PDF)
Molecular formula strings (CSV)

## Abbreviations

ACN: acetonitrile
AcOH: acetic acid
CCCP: carbonyl cyanide *m*-chlorophenyl hydrazone
CMC: critical micellar concentration
DCC: dicyclohexylcarbodiimide
DCM: dichloromethane
DDM: dodecyl maltoside
DIC: diisopropylcarbdodiimide
DIEA: diisopropylethylamine
DM: decyl maltosid
DMF: dimethylformamide
DMSO: dimethylsulfoxide
DTT: dithiothreitol
FA: formic acid
FRET: Förster resonance energy transfer
HATU: 2-(1H-(7-azabenzotriazol-1-yl))-1,1,3,3-tetramethyluronium hexafluorophosphate
HBTU: 2-(1H-benzotriazol-1-yl)-1,1,3,3-tetramethyluronium hexafluorophosphate
HOBt: 1-hydroxybenzotriazol
iPrOH: isopropyl alcohol
NG: nonylglucoside
PyBrOP: *tris* (N-pyrolidyl) phosphoryl bromide hexafluorophosphate
TCEP: *tris*(2-carboxyethyl)phosphine
TFA: trifluoroacetic acid
TFE: trifluoroethanol
TM: transmembrane
pTSA.H_2_O: p-toluenesulfonic acid monohydrate
