# Supplementary material for: Chemical Blockage of the Mitochondrial Rhomboid Protease PARL by Novel Ketoamide Inhibitors Reveals Its Role in PINK1/Parkin-Dependent Mitophagy
Source: J Med Chem. 2022 Dec 21;66(1):251–65. doi: 10.1021/acs.jmedchem.2c01092 (PMC9841525; doi:10.1021/acs.jmedchem.2c01092)
Supplement: Supplementary file 1 — jm2c01092_si_001.pdf [file jm2c01092_si_001.pdf]

**SUPPORTING INFORMATION**

# Chemical blockage of the mitochondrial rhomboid protease PARL by novel ketoamide inhibitors reveals its role in PINK1/Parkin-dependent mitophagy.

Edita Poláchová [1,3,#], Kathrin Bach [1,2,#], Elena Heuten [7,8,#], Stancho Stanchev [1], Anežka Tichá [1], Philipp Lampe [4], Pavel Majer [1], Thomas Langer [4,5,6], Marius K. Lemberg [7,8]* and Kvido Strisovsky [1]*

[1]Institute of Organic Chemistry and Biochemistry of the Czech Academy of Science, Flemingovo n. 2, Prague, 160 00, Czech Republic

[2]Department of Molecular Genetics, Faculty of Science, Charles University, Viničná 5, Prague, 128 44, Czech Republic

[3]First Faculty of Medicine, Charles University, Kateřinská 32, Prague, 121 08, Czech Republic

[4]Institute for Genetics and Cologne Excellence Cluster on Cellular Stress Responses in Aging-Associated Diseases (CECAD), Medical Faculty, University of Cologne, Joseph-Stelzmann-Strasse 52, Cologne, 50931, Germany.

[5]Center for Molecular Medicine (CMMC), Medical Faculty, University of Cologne, Joseph-Stelzmann-Strasse 52, Cologne, 50931, Germany.

[6]Max-Planck-Institute for Biology of Ageing, Joseph-Stelzmann-Str. 9b, Cologne, 50931, Germany

[7]Center for Molecular Biology of Heidelberg University (ZMBH), DKFZ-ZMBH Alliance, Im Neuenheimer Feld 282, 69120 Heidelberg, Germany.

[8]Center for Biochemistry and Cologne Excellence Cluster on Cellular Stress Responses in Aging-Associated Diseases (CECAD), Medical Faculty, University of Cologne, Joseph-Stelzmann-Strasse 52, Cologne, 50931, Germany.

[#]equal contribution

*corresponding authors: kvido.strisovsky@uochb.cas.cz, m.lemberg@uni-koeln.de

## Contents of SI

Supplementary Scheme1

Supplementary Figure S1

Supplementary Figure S2

Compound characterization data

Molecular formula strings

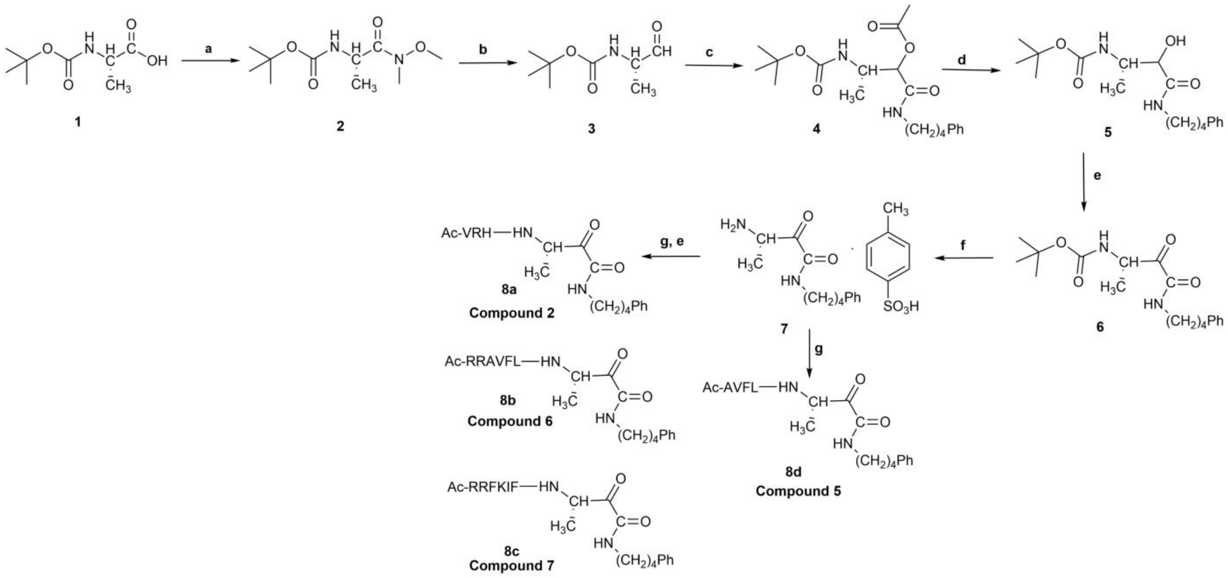

**Scheme 1.** Synthetic scheme of peptidyl-α-ketoamide inhibitors**.**

**Reagents and conditions:** (a) $NH(CH_3)OCH_3$. HCl, DCM, NMM, 16 h, r.t., 92 %; (b) $LiAlH_4$, THF, 90 min, 0 – 25$^0$C , 60 %; (c) Ph-$(CH_2)_4$-NC, AcOH, DCM dry, 24h, r.t.; (d) LiOH, water/$CH_3OH$ (17:83), r. t., 70 min., 50 % in two steps; (e) Dess-Martin periodinane, 4 h, DCM dry, r.t., 90 %; (f) p-TSA. $H_2O$, ACN, sonication 4 x 3 min, r.t.; (g) Ac-VR(Pbf)H(Trt)-OH(8a), Ac-$[R(Pbf)]_2$AVFL-OH (8b), Ac-$[R(Pbf)]_2$FK(Boc)IF-OH (8c), Ac-AVFL-OH (8d), PyBrOP, HOBt, DIEA, DCM, overnight, 0$^0$C - r.t.; (e) TFA/triisopropylsilane/water = 95:2.5:2.5, 1.5h, r.t, 7 – 20 % in the last three steps.

**Fig. S1:** Mass spectrometric characterisation of compound **1** cleavage by GlpG

**(A)** Compound 1 was exposed to the indicated treatments and separated via reversed phase HPLC. The main peak areas around 31-33 min and 51.5 to 60 min (dashed lines) were analysed by ESI mass spectrometry. The resulting mass spectra are show in panel **(B)**, and exact masses of the expected compounds and their hydrolysis products are displayed below. The mass spectra in panel **(B)** originate from the sample "Ac-RVRHA-4mc + GlpG" (trace marked red in panel **(A)**).

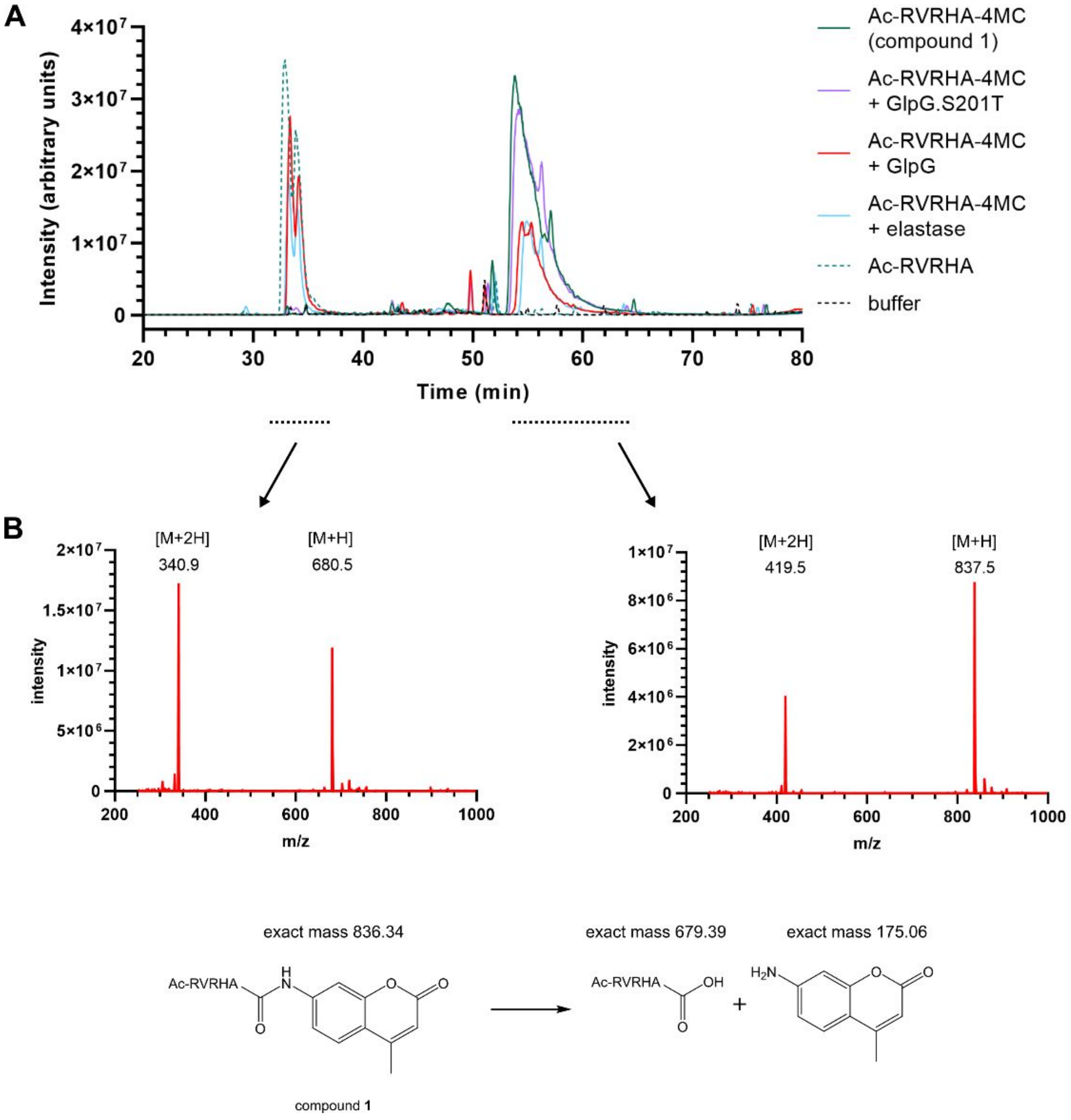

**Fig. S2:** Mitochondrial membrane potential is not affected by compound **5**.

JC-1 membrane potential assay shows that compound **5** does not depolarize the mitochondrial membrane potential. HEK293T cells were treated for at least three hours with 5 µM compound **5** or CCCP or DMSO, and then stained with JC-1 and Hoechst (blue) for nuclei. Images were acquired via live cell imaging while continuing treatment. Images are maximum intensity z-stack projections with scale bars of 20 µm. Significant changes in ratio, indicative of membrane potential, are annotated between DMSO and CCCP as well as CCCP and compound **5** treatment (*$p \leq 0.05$, **$p \leq 0.01$, unpaired t-test; means ± SEM, n=3, N(DMSO) = 1584, N(CCCP) = 1500, N(compound **5**) = 1358).

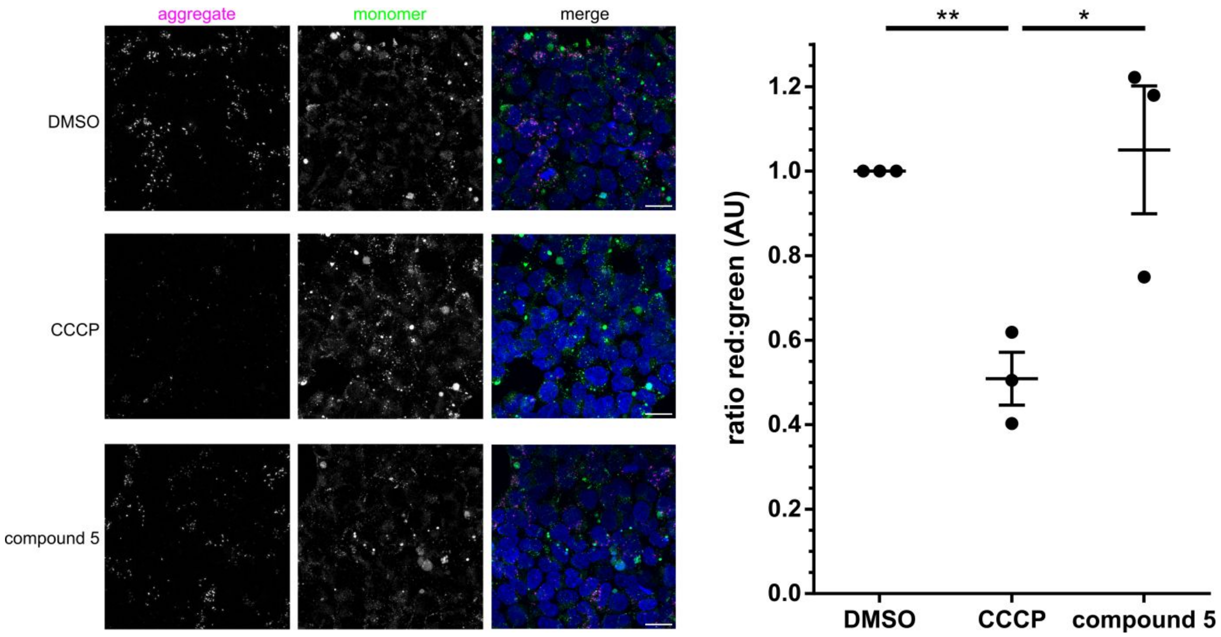

**Compound characterisation data**

**Compound 1; Ac-RVRHA-4mc**

Purified, using gradient 10 – 40% B. Yield: 25 mg.

$^1$H NMR (400 MHz, DMSO) δ (ppm) = 14.20 (s, 1H, imidazole), 10.59 – 10.39 (m, 1H, CONH, aromatic), 9.03 – 8.93 (m, 1H, aromatic), 8.46 – 8.34 (m, 1H, CONH), 8.29 – 8.22 (m, 1H, CONH), 8.21 – 8.13 (m, 2H, CONH), 7.81 (dd, J = 11.2, 2.1 Hz, 1H, CONH), 7.73 (d, J = 8.7 Hz, 1H, aromatic), 7.69 – 7.53 (m, 3H, aromatic), 7.46 (td, J = 8.6, 2.1 Hz, 1H, NH), 7.41 – 7.33 (m, 1H, NH), 6.31 – 6.27 (m, 1H, CH), 4.71 – 4.56 (m, 1H, CH), 4.40 (dt, J = 11.1, 6.8 Hz, 1H, CH), 4.32 – 4.07 (m, 3H, CH), 3.21 – 2.88 (m, 6H, $CH_2$), 2.41 (s, 3H, $CH_3$), 1.94 (dd, J = 14.4, 7.8 Hz, 1H, CH), 1.86 (d, J = 8.7 Hz, 3H, acetyl), 1.71 – 1.40 (m, 8H, $CH_2$), 1.33 (t, J = 7.4 Hz, 3H, $CH_3$), 0.79 (dt, J = 11.6, 7.1 Hz, 6H, $CH_3$).

$^{13}$C NMR (101 MHz, DMSO) δ (ppm) = 172.30, 171.85, 170.30, 169.90, 160.50, 158.84, 157.23, 154.12, 153.57, 142.60, 134.14, 129.58, 126.43, 115.72, 112.86, 106.20, 64.96, 63.17, 60.60, 57.67, 52.91, 51.75, 31.18, 29.13, 25.61, 22.89, 19.58, 18.45.

HR-MS: Calculated monoisotopic mass for $C_{38}H_{56}N_{14}O_8$ 836.44, found [M+H$^+$] $C_{38}H_{57}N_{14}O_8$ = 837.448.

**Compound 2; Ac-VRHA-COCONH-($CH_2$)$_4$-Ph**

Purified, using gradient 10 – 40% B. Yield: 4 mg.

$^1$H NMR (401 MHz, DMSO) δ (ppm) = 14.20 (s, 1H, imidazole), 8.97 (s, 1H, COCONH), 8.71 (t, J = 6.0 Hz, 1H, aromatic), 8.41 (d, J = 6.5 Hz, 1H, CONH), 8.20 (d, J = 8.1 Hz, 1H, CONH), 8.09 (d, J = 7.3 Hz, 1H, CONH), 7.88 (d, J = 8.2 Hz, 1H, CONH), 7.55 (d, J = 5.3 Hz, 1H, aromatic), 7.34 (s, 1H, NH), 7.30 – 7.23 (m, 3H, aromatic + NH), 7.20 – 7.13 (m, 3H, aromatic), 5.06 – 4.96 (m, 1H, CH), 4.67 – 4.56 (m, 1H, CH), 4.25 – 4.16 (m, 1H, CH), 4.09 (t, J = 7.6 Hz, 1H, CH), 3.14 (q, J = 6.4 Hz, 2H, $CH_2$), 3.11 – 3.00 (m, 3H, $CH_2$ + CH), 2.91 (dd, J = 15.4, 8.2 Hz, 1H, $CH_2$), 2.61 – 2.53 (m, 2H, $CH_2$), 1.94 – 1.89 (m, 1H, CH), 1.87 (s, 3H, acetyl), 1.59 – 1.34 (m, 8H, $CH_2$), 1.24 (d, J = 7.3 Hz, 3H, $CH_3$), 0.82 (d, J = 6.8 Hz, 6H, $CH_3$).

$^{13}$C NMR (101 MHz, DMSO) δ (ppm) = 196.76, 171.5, 169.5, 158.3, 156.65, 142.01, 138.3, 136.2, 133.7, 128.1, 125.7, 63.6, 58.0, 53.2, 50.9, 48.6, 34.7, 30.13, 28.29, 25.06, 22.45, 18.31, 15.6

HR-MS: Calculated monoisotopic mass for $C_{33}H_{50}N_{10}O_6$ 682.4, found [M+H$^+$] $C_{33}H_{51}N_{10}O_6$ = 683.399.

**Compound 3; Ac-AVFLA-4mc**

Purified, using gradient 30 – 80% B. Yield: 13.7 mg

$^{1}H$ NMR (401 MHz, DMSO) δ (ppm) = 10.38 (s, 1H, CONH aromatic), 8.21 (d, J = 6.7 Hz, 1H, CONH), 8.07 – 7.94 (m, 3H, CONH), 7.79 – 7.70 (m, 2H, aromatic), 7.67 (d, J = 8.8 Hz, 1H, aromatic), 7.49 (dd, J = 8.7, 2.0 Hz, 1H, CONH), 7.22 (d, J = 4.3 Hz, 4H, aromatic), 7.19 – 7.12 (m, 1H, aromatic), 6.27 (d, J = 1.2 Hz, 1H, CH), 4.57 (td, J = 9.2, 4.5 Hz, 1H, CH), 4.44 – 4.33 (m, 2H, CH), 4.28 (t, J = 7.2 Hz, 1H, CH), 4.13 – 4.04 (m, 1H, CH), 3.01 (dd, J = 14.0, 4.4 Hz, 1H, $CH_2$), 2.78 (dd, J = 14.1, 9.6 Hz, 1H, $CH_2$), 2.40 (s, 3H, $CH_3$), 1.94 – 1.86 (m, 1H, CH), 1.82 (s, 3H, acetyl), 1.61 (dd, J = 13.4, 6.7 Hz, 1H, CH), 1.46 (t, J = 7.2 Hz, 2H, $CH_2$), 1.33 (d, J = 7.1 Hz, 3H, $CH_3$), 1.12 (d, J = 7.1 Hz, 3H, $CH_3$), 0.89 (d, J = 6.6 Hz, 3H, $CH_3$), 0.84 (d, J = 6.5 Hz, 3H, $CH_3$), 0.73 (dd, J = 6.7, 3.4 Hz, 6H, $CH_3$).

$^{13}C$ NMR (101 MHz, DMSO) δ (ppm) = 172.31, 171.84, 171.73, 170.84, 170.67, 169.07, 160.00, 153.65, 153.09, 142.27, 137.66, 129.06, 127.98, 126.18, 125.97, 115.11 (d, J = 12.3 Hz), 112.31, 105.61, 57.53, 53.53, 50.82, 49.27, 48.16, 37.29, 30.59, 24.01, 23.14, 22.44, 21.54, 19.09, 17.99, 17.70.

HR-MS: Calculated monoisotopic mass for $C_{38}H_{50}N_6O_8$ 718.37, found [M+H$^+$] $C_{38}H_{51}N_6O_8$ = 719.38 and [M+Na$^+$] $C_{38}H_{50}N_6O_8Na$ 741.358.

**Compound 4 ; Ac-RRRAVFLA-4mc**

Purified, using gradient 15 – 50% B. Yield: 10.4 mg

$^{1}H$ NMR (401 MHz, DMSO) δ (ppm) = 10.44 (s, 1H, CONH, aromatic), 8.25 (d, J = 6.9 Hz, 1H, CONH), 8.09 (d, J = 7.5 Hz, 2H, CONH), 8.06 – 7.98 (m, 3H, CONH), 7.84 – 7.69 (m, 2H, aromatic), 7.65 – 7.53 (m, 2H, CONH), 7.49 (dd, J = 8.7, 2.1 Hz, 1H, aromatic), 7.28 – 7.11 (m, 6H, aromatic + NH), 6.28 (d, J = 1.1 Hz, 1H, CH), 4.59 (td, J = 8.8, 4.6 Hz, 1H, CH), 4.44 – 4.33 (m, 2H, CH), 4.33 – 4.19 (m, 4H, CH), 4.15 – 4.08 (m, 1H, CH), 3.08 (t, J = 6.1 Hz, 6H, $CH_2$), 2.41 (d, J = 1.3 Hz, 3H, $CH_3$), 1.91 (q, J = 6.8 Hz, 1H, CH), 1.87 (s, 3H, acetyl), 1.72 – 1.59 (m, 3H, $CH_2$ + CH), 1.57 – 1.40 (m, 10H, $CH_2$), 1.34 (d, J = 7.1 Hz, 2H, $CH_2$), 1.14 (d, J = 7.0 Hz, 3H, $CH_3$), 0.89 (d, J = 6.6 Hz, 3H, $CH_3$), 0.85 (d, J = 6.5 Hz, 3H, $CH_3$), 0.80 – 0.70 (m, 6H, $CH_3$).

$^{13}C$ NMR (101 MHz, DMSO) δ (ppm) = 193.95, 172.34, 168.02, 166.26, 157.16, 153.99, 152.66, 140.24, 129.57, 128.44, 121.26, 120.51, 118.88, 115.94, 100.23, 62.60, 57.87, 53.94, 48.66, 31.27, 23.63, 22.92, 21.98, 19.56, 18.36.

HR-MS: Calculated monoisotopic mass for $C_{56}H_{86}N_{18}O_{11}$ 1186.67, found [M+H]$^{2+}$ $C_{56}H_{88}N_{18}O_{11}$ = 594.343 and [M+H]$^{3+}$ $C_{56}H_{89}N_{18}O_{11}$ 396.565.

**Compound 5; Ac-AVFLA-COCONH-$(CH_2)_4$-Ph**

Purified, using gradient 30 – 80% B. Yield: 20 mg

$^{1}H$ NMR (400 MHz, DMSO) δ (ppm) = 8.69 (t, J = 6.2 Hz, 1H, COCONH), 8.30 (d, J = 6.0 Hz, 1H, CONH), 8.03 (d, J = 7.3 Hz, 1H, CONH), 7.94 (t, J = 8.8 Hz, 2H, CONH), 7.64 (d, J = 8.8 Hz, 1H, CONH), 7.29 – 7.12 (m, 11H, aromatic), 4.99 – 4.88 (m, 1H, CH), 4.61 – 4.50 (m, 1H, CH), 4.35 (d, J = 5.9 Hz, 1H, CH), 4.30 – 4.23 (m, 1H, CH), 4.11 – 4.02 (m, 1H, CH), 3.13 (d, J = 7.0 Hz, 1H, $CH_2$), 3.03 – 2.95 (m, 1H, $CH_2$), 2.77 (dd, J = 14.0, 9.5 Hz, 1H, $CH_2$), 2.56 (t, J = 7.3 Hz, 2H, $CH_2$), 1.94 – 1.85 (m, 1H, CH), 1.82 (s, 3H, acetyl), 1.66 – 1.36 (m, 6H, $CH_2$), 1.25 (d, J = 7.3 Hz, 3H, $CH_3$), 1.12 (d, J = 7.1 Hz, 3H, $CH_3$), 0.92 – 0.77 (m, 6H, $CH_3$), 0.73 (dd, J = 6.7, 2.3 Hz, 6H, $CH_3$).

$^{13}C$ NMR (101 MHz, DMSO) δ (ppm) = 197.03, 172.23, 170.57, 169.02, 168.23, 165.56, 160.55, 142.03, 137.65, 129.08, 128.23, 127.95, 126.15, 125.62, 69.77, 57.48, 53.43, 49.32, 48.09, 34.68, 30.58, 28.28, 22.99, 22.42, 21.69, 19.06, 17.82, 15.47.

HR-MS: Calculated monoisotopic mass for $C_{39}H_{56}N_6O_7$ 720.42, found [M+H$^+$] $C_{39}H_{57}N_6O_7$ = 721.428 and [M+Na$^+$] $C_{39}H_{56}N_6O_7Na$ 743.410.

**Compound 6; Ac-RRAVFLA-COCONH-($CH_2$)$_4$-Ph**

Purified, using gradient 30 – 80% B. Yield: 25.5 mg

$^{1}H$ NMR (401 MHz, DMSO) δ (ppm) = 8.74 – 8.65 (m, 1H, COCONH), 8.16 – 7.90 (m, 5H, CONH), 7.77 – 7.67 (m, 1H, CONH), 7.61 – 7.44 (m, 2H, CONH), 7.30 – 7.11 (m, 13H, aromatic + NH), 5.00 – 4.88 (m, 1H, CH), 4.55 (t, J = 6.7 Hz, 1H, CH), 4.27 (d, J = 6.7 Hz, 4H, CH), 4.09 (d, J = 9.2 Hz, 1H, CH), 3.16 – 2.95 (m, 6H, $CH_2$), 2.81 – 2.72 (m, 1H, $CH_2$) 2.56 (t, J = 7.6 Hz, 2H, $CH_2$), 1.89 (d, J = 9.0 Hz, 1H, CH), 1.85 (s, 3H, acetyl), 1.72 – 1.59 (m, 2H, $CH_2$), 1.58 – 1.36 (m, 12H, $CH_2$), 1.25 (d, J = 7.3 Hz, 3H, $CH_3$), 1.13 (d, J = 7.1 Hz, 3H, $CH_3$), 0.92 – 0.79 (m, 6H, $CH_3$), 0.79 – 0.68 (m, 6H, $CH_3$).

$^{13}C$ NMR (101 MHz, DMSO) δ (ppm) = 198.08, 189.78, 178.43, 176.66, 174.34, 170.63, 159.30, 156.67, 143.1, 128.29, 125.67, 65.99, 57.65, 57.39, 51.77, 30.78, 29.08, 28.31, 24.98, 22.46, 19.11, 17.93.

HR-MS: Calculated monoisotopic mass for $C_{51}H_{80}N_{14}O_9$ 1032.62, found [M+H]$^{2+}$ $C_{51}H_{82}N_{14}O_9$ = 517.319.

**Compound 7; Ac-RRFKIF-COCONH-($CH_2$)$_4$-Ph**

Purified, using gradient 15 – 50% B. Yield: 4.5 mg

$^{1}H$ NMR (401 MHz, DMSO) δ (ppm) = 8.75 (t, J = 6.3 Hz, 1H, COCONH), 8.48 (t, J = 7.3 Hz, 1H, CONH), 8.26 (s, 1H, CONH), 8.08 (d, J = 7.7 Hz, 1H, CONH), 7.98 (d, J = 8.4 Hz, 1H, CONH), 7.70 (s, 3H, NH3), 7.54 (d, J = 9.7 Hz, 1H, CONH), 7.36 – 7.08 (m, 18H, aromatic), 5.36 - 5.25 (m, 1H, CH), 4.60 – 4.48 (m, 1H, CH), 4.25 (dd, J = 20.1, 6.1 Hz, 3H, CH), 3.23 – 2.93 (m, 8H, $CH_2$), 2.85 – 2.63

(m, 4H, $CH_2$), 2.61 – 2.53 (m, 2H, $CH_2$), 1.84 (s, 3H, acetyl), 1.72 – 1.33 (m, 16H, $CH_2$), 1.32 – 1.19 (m, 3H, $CH_3$), 0.90 – 0.69 (m, 6H, $CH_3$).

HR-MS: Calculated monoisotopic mass for $C_{55}H_{82}N_{14}O_8$ 1066.64, found $[M+H]^+$ $C_{55}H_{83}N_{14}O_8$ = 1067.650 and $[M+H]^{2+}$ $C_{55}H_{84}N_{14}O_8$ 534.329.
